# Population Pharmacokinetics and Exposure-Response Relationships of Baloxavir Marboxil in Influenza Patients at High Risk of Complications

**DOI:** 10.1128/AAC.00119-20

**Published:** 2020-06-23

**Authors:** Hiroki Koshimichi, Sylvie Retout, Valerie Cosson, Vincent Duval, Stefan De Buck, Yoshiyuki Tsuda, Toru Ishibashi, Toshihiro Wajima

**Affiliations:** a Clinical Pharmacology & Pharmacokinetics, Project Management Department, Shionogi & Co., Ltd., Osaka, Japan; b Roche Pharma Research and Early Development, Pharmaceutical Sciences, Roche Innovation Center Basel, Switzerland; c Certara, Data Science Services, Basel, Switzerland

**Keywords:** baloxavir marboxil, cap-dependent endonuclease inhibitor, influenza, population pharmacokinetics, S-033188, exposure-response, high risk of influenza complications

## Abstract

Baloxavir marboxil, a prodrug of cap-dependent endonuclease inhibitor baloxavir acid, reduces the time to improvement of influenza symptoms in patients infected with type A or B influenza virus. To characterize its pharmacokinetics, a population pharmacokinetic model for baloxavir acid was developed using 11,846 plasma concentration data items from 1,827 subjects, including 2,341 plasma concentration data items from 664 patients at high risk of influenza complications. A three-compartment model with first-order elimination and first-order absorption with lag time well described the plasma concentration data. Body weight and race were found to be the most important factors influencing clearance and volume of distribution. The exposures in high-risk patients were similar to those in otherwise healthy patients, and no pharmacokinetic difference was identified regarding any risk factors for influenza complications. Exposure-response analyses were performed regarding the time to improvement of symptoms and the reduction in the influenza virus titer in high-risk patients. The analyses suggested that body weight-based dosage, 40 mg for patients weighing <80 kg and 80 mg for patients weighing ≥80 kg, can shorten the time to improvement of influenza symptoms and reduce virus titer for both type A and B influenza virus regardless of the exposure levels of the high-risk patients as well as for the otherwise healthy influenza patients. The results of our population pharmacokinetic and exposure-response analyses in patients with risk factors of influenza complications should provide useful information on the pharmacokinetic and pharmacodynamic characteristics of baloxavir marboxil and also for the optimization of dose regimens.

## Abstract (precis)

Baloxavir marboxil, a prodrug of cap-dependent endonuclease inhibitor baloxavir acid, reduces the time to improvement of influenza symptoms in patients infected with type A or B influenza virus. To characterize its pharmacokinetics, a population pharmacokinetic model for baloxavir acid was developed using 11,846 plasma concentration data items from 1,827 subjects, including 2,341 plasma concentration data items from 664 patients at high risk of influenza complications. A three-compartment model with first-order elimination and first-order absorption with lag time well described the plasma concentration data.

## INTRODUCTION

Baloxavir marboxil (product code S-033188, Xofluza) is a prodrug of baloxavir acid, a potent and selective inhibitor of cap-dependent endonuclease necessary for the replication of both influenza A and B viruses. Baloxavir marboxil has been approved in Japan and the United States for the treatment of adults and adolescents (at least 12 years old) with acute uncomplicated influenza (Xofluza package insert).

After single oral administration, baloxavir marboxil is metabolized to baloxavir acid mainly by arylacetamide deacetylase (AADAC) in the intestine and liver, and baloxavir acid is eliminated from the plasma via a metabolism by UDP-glucuronosyltransferase 1A3 (UGT1A3) in the liver, with minor contribution from cytochrome P450 3A4 (CYP3A4) (1). In a thorough QTc study (see Table S1 in the supplemental material), in which 39 males and 24 females were assessed in the pharmacokinetic analyses in the same study, time to maximum plasma concentration was slightly longer in females (median at 80 mg: 4 h) compared to male subjects (median at 80 mg: 3 h), indicating a potential gender effect on absorption.

A randomized, double-blind, controlled phase 2 study in otherwise healthy adults (hereafter, phase 2 OwH study) and a phase 3 study in otherwise healthy adults and adolescents (CAPSTONE-1; hereafter, phase 3 OwH study) were conducted with patients suffering from acute uncomplicated influenza (2). Baloxavir marboxil significantly reduced the time to alleviation of influenza (the primary endpoint) compared to placebo. Interestingly, on the secondary endpoint (viral load reduction), baloxavir marboxil showed a significantly faster reduction in viral load compared to not only the placebo treatment group but also the oseltamivir treatment groups. Using two clinical studies with otherwise healthy influenza patients and 10 phase 1 studies, population pharmacokinetic analysis and exposure-response analysis of baloxavir acid were conducted (1). Body weight and race (Asian or non-Asian) were identified as the most significant covariates on the apparent total clearance (CL/*F*) and the apparent central volume of distribution (*V_c_*/*F*) of baloxavir acid. In addition, a hepatic function marker (alanine aminotransferase [ALT]) on CL/*F*, gender on absorption rate constant (*K_a_*), and food intake on bioavailability (*F*) were identified as significant covariates. Exposure-response analyses suggested that the treatment of baloxavir marboxil for otherwise healthy influenza patients significantly shortened the time to alleviation of symptoms and reduced viral load compared to the placebo treatment group, with an apparent near maximum response over a wide dose and concentration range in both Asian and non-Asian patients, despite the race effect on the pharmacokinetics of baloxavir acid. In addition, no apparent safety concerns were observed, suggesting that baloxavir acid has a wide therapeutic window.

In addition to the two clinical trials with otherwise healthy influenza patients, a clinical study for treatment of influenza in patients at high risk of complications (CAPSTONE-2; hereafter, phase 3 HR study) was conducted as a double-blind, placebo- and oseltamivir-controlled study with adults and adolescents from 2016 to 2018 (3). The definition of high risk of influenza complications was adapted from Centers for Disease Control and Prevention (CDC) criteria (http://www.cdc.gov/flu/about/disease/high_risk.htm), which included influenza with asthma or chronic lung disease, age of 65 years, or morbid obesity (see reference 3 for more details). The median time to improvement of influenza symptoms (the primary endpoint) in the baloxavir treatment group was 29.1 h shorter than that in the placebo treatment group (73.2 h versus 102.3 h) and similar to that in the oseltamivir treatment group (81.0 h). The treatment with baloxavir marboxil demonstrated a significantly larger viral load decline compared to those in the placebo and oseltamivir treatment groups, with median reductions from baseline in virus titer on day 2 of 3.45 log_10_(TCID_50_/ml) (where TCID_50_ is 50% tissue culture infectious dose) in the baloxavir treatment group versus 1.80 log_10_(TCID_50_/ml) in the oseltamivir treatment group and 1.20 log_10_(TCID_50_/ml) in the placebo treatment group.

Patient populations infected with influenza at high risk of complications are different from otherwise healthy influenza patients, primarily in terms of risk factors, which are heterogeneous in nature. It cannot be ruled out beforehand whether any such factors may present a relevant covariate on the pharmacokinetics and/or exposure-effect relationship of baloxavir acid. In the phase 3 HR study, a total of 2,341 additional plasma concentrations in 664 patients were obtained from different patient populations along with the efficacy data. The aim of this study was to construct a population pharmacokinetic model based on the pooled data from all clinical trials, including the phase 3 HR study, and to assess the effects of background characteristics, including risk factors of influenza complications, on the pharmacokinetics of baloxavir acid. In addition, exposure-response relationships were investigated in the patients at high risk of influenza complications. This paper is the first report which includes the population pharmacokinetic analysis and exposure-response analysis in influenza patients at high risk of developing influenza-related complications, which may result in hospitalization or death, for not only baloxavir marboxil but also other drugs against influenza.

## RESULTS

### Population pharmacokinetic analysis.

Background characteristics, i.e., demographic, clinical laboratory test results, and disease-related parameters, from 13 clinical studies used for the population pharmacokinetic analyses are summarized in Table 1. The model-building process is shown in Table S2. A three-compartment model with first-order elimination and first-order absorption with lag time was selected as the structural model (4). Inferential assessments were conducted based on the full model (model no. 407) to quantify the importance of the detected covariate effects on the pharmacokinetics. The ratios of parameters of interest calculated at the lower and upper range of covariate distributions in the database relative to the reference value (median for continuous covariates and mode for categorical covariates) and their 95% confidence intervals (CIs) were estimated and are shown in Fig. S1. The covariates with a ratio considered to be small (point estimates of the ratios close to 1 and their 95% CIs within or close to the range of 0.80 to 1.25) were removed from the model. The final model (model no. 523) includes the effect of body weight on CL/*F* and intercompartment clearances (*Q*_1_/*F* and *Q*_2_/*F*) (the same exponent), the effect of body weight on *V_c_*/*F* and peripheral volume of distributions (*V_p1_*/*F* and *V_p2_*/*F*) (the same exponent), the effects of race (Asian or non-Asian) on CL/*F* and *V_c_*/*F*, and the effect of gender on *K_a_* (Table 2). Relationships of interindividual variabilities (ηs) with covariates are shown in Fig. S2. The tendencies of ηs for CL/*F* and *V_c_*/*F* against body weight and race (Asian or non-Asian) visible in the base model (Fig. S3 and Table S3) disappeared in the final model (Fig. S2), indicating that the final model could appropriately explain the effects of background characteristics on the pharmacokinetics of baloxavir marboxil.

**TABLE 1:** Subject characteristics and pharmacokinetic parameters on which the covariates were tested^*a*^

Characteristic	Value for:				Pharmacokinetic parameter(s)
	Healthy subjects		Patients		
	Asian^*b*^ (*n* = 231)	Non-Asian^*b*^ (*n* = 46)	Asian^*b*^ (*n* = 844)	Non-Asian^*b*^ (*n* = 706)	
Age (yr)					CL/*F*, *V_c_*/*F*, *K_a_*
Mean (SD)	29.1 (7.6)	49.2 (11.8)	40.0 (15.9)	44.3 (17.6)	
Median (range)	27 (20–49)	48 (26–70)	38 (12–85)	45 (12–84)	
No. of male/female subjects	207/24	37/9	487/357	289/419	CL/*F*, *V_c_*/*F*, *K_a_*
Ht (cm)					
Mean (SD)	170.3 (6.7)	172.3 (9.8)	165.4 (9.1)	166.8 (10.1)	
Median (range)	171.0 (148.3–187.5)	173.0 (153.0–205.0)	166.0 (137.5–192.0)	166.1 (121.9–195.6)	
Body wt (kg)					CL/*F*, *Q*_1_/*F*, *Q*_2_/*F*, *V_c_*/*F*, *V_p1_*/*F*, *V_p2_*/*F*
Mean (SD)	61.7 (6.9)	83.1 (13.9)	63.4 (13.5)	83.1 (21.7)	
Median (range)	61.1 (46.0–77.2)	82.7 (56.7–118.9)	61.9 (36.0–118.4)	80.0 (40.8–217.3)	
BMI (kg/m^2^)					CL/*F*, *V_c_*/*F*
Mean (SD)	21.2 (1.5)	27.9 (3.8)	23.0 (3.9)	29.8 (7.0)	
Median (range)	21.1 (18.5–24.9)	28.3 (21.0–37.8)	22.3 (15.3–37.8)	28.6 (16.5–69.4)	
AST (U/liter)					CL/*F*
Mean (SD)	17.1 (4.0)	23.9 (7.8)	25.1 (19.8)	25.6 (22.3)	
Median (range)	17 (9–32)	23 (14–62)	21 (10–428)	21 (11–355)	
ALT (U/liter)					CL/*F*
Mean (SD)	15.9 (7.1)	23.4 (8.8)	23.4 (20.7)	26.9 (31.0)	
Median (range)	14 (6–39)	22 (10–50)	17 (6–320)	20 (6–552)	
eGFR_adj_ (ml/min/1.73m^2^)					CL/*F*
Mean (SD)	87.8 (11.4)	114.0 (26.3)	78.9 (20.8)	94.6 (26.6)	
Median (range)	87.4 (63.3–119.8)	114.9 (60.4–176.8)	76.3 (27.4–173.9)	92.2 (14.2–213.3)	
eGFR_abs_ (ml/min)					CL/*F*
Mean (SD)	86.5 (11.7)	130.2 (28.7)	77.0 (20.2)	105.9 (31.4)	
Median (range)	86.0 (60.0–121.6)	130.3 (76.5–190.1)	74.9 (24.8–173.0)	102.6 (17.4–271.8)	
CL_CR_ (ml/min)					CL/*F*
Mean (SD)	111.1 (15.3)	140.6 (31.8)	101.4 (28.4)	125.2 (48.3)	
Median (range)	110.3 (77.7–158.8)	138.2 (84.4–209.2)	99.6 (26.3–225.6)	119.5 (21.7–431.7)	
No. of patients in OwH study/HR study	0/0	0/0	658/186	228/478	
No. (%) of influenza virus-infected patients	0 (0)	0 (0)	807 (95.6)	319 (45.2)	
No. (%) of subjects by food condition^*c*^					*F*
Fasted	231 (100.0)	46 (100.0)	236 (28.0)	299 (42.4)	
Intermediate	0 (0.0)	0 (0.0)	275 (32.6)	165 (23.4)	
Fed	0 (0.0)	0 (0.0)	333 (39.5)	242 (34.3)	
No. (%) of subjects with asthma or chronic lung disease	0 (0.0)	0 (0.0)	62 (7.3)	219 (31.0)	CL/*F*
No. (%) of subjects with endocrine disorders	0 (0.0)	0 (0.0)	59 (7.0)	148 (21.0)	CL/*F*
No. (%) of subjects with neurological and neurodevelopmental disorders	0 (0.0)	0 (0.0)	9 (1.1)	29 (4.1)	CL/*F*
No. (%) of subjects with heart disease	0 (0.0)	0 (0.0)	16 (2.0)	59 (8.4)	CL/*F*
No. (%) of subjects ≥65 yrs of age	0 (0.0)	5 (10.9)	75 (8.9)	106 (15.0)	CL/*F*
No. (%) of subjects with metabolic disorders	0 (0.0)	0 (0.0)	36 (4.3)	26 (3.7)	CL/*F*
No. (%) of subjects with morbid obesity (BMI ≥ 40)	0 (0.0)	0 (0.0)	0 (0.0)	73 (10.3)	CL/*F*

a BMI, body mass index; AST, aspartate aminotransferase; ALT, alanine aminotransferase; eGFR_adj_, body surface area-adjusted estimated glomerular filtration rate; eGFR_abs_, absolute estimated glomerular filtration rate; CL_CR_, creatinine clearance.

b Race (Asian or non-Asian) was also tested as a covariate on CL/*F* and *V_c_*/*F*.

c Fasted, dosing ≥4 h before and after food intake; intermediate, dosing within 2 to 4 h before or after food intake; fed, dosing <2 h before or after food intake.

**TABLE 2:** Population pharmacokinetic parameter estimates in the final model^*a*^

Parameter	Final model		Bootstrap estimates	
	Estimate	%RSE	Median	95% CI
Pharmacokinetic parameters				
CL/*F* (liters/h)	10.8	1.8	10.8	10.4–11.3
*V_c_*/*F* (liters)	565	3.0	565	528–604
*Q*_1_/*F* (liters/h)	12.4	6.9	12.4	10.3–14.2
*V_p1_*/*F* (liters)	141	3.1	141	130–152
*Q*_2_/*F* (liters/h)	1.43	4.3	1.42	1.25–1.59
*V_p2_*/*F* (liters)	139	2.4	139	131–147
*K_a_* (liters/h)	1.03	6.3	1.03	0.905–1.20
Lag time (h)	0.345	3.3	0.344	0.323–0.371
Effect of body wt on CL/*F*, *Q*_1_/*F*, and *Q*_2_/*F*	0.362	10.4	0.363	0.278–0.445
Effect of body wt on *V_c_*/*F*, *V_p1_*/*F*, and *V_p2_*/*F*	0.833	5.0	0.833	0.743–0.926
Effect of race (Asian) on CL/*F*	0.519	2.2	0.519	0.495–0.544
Effect of race (Asian) on *V_c_*/*F*	0.564	3.6	0.564	0.523–0.609
Effect of gender on *K_a_*	0.682	9.1	0.679	0.566–0.819
% CV for IIV for CL/*F* (sh_ηp) (%)	41.1 (4.4)	4.4	41.1	39.3–42.9
% CV for IIV for *V_c_*/*F* (sh_ηp) (%)	62.7 (10.9)	4.6	62.6	59.7–65.4
% CV for IIV for *V_p1_*/*F* (sh_ηp) (%)	29.3 (61.5)	16.7	29.3	23.9–34.3
% CV for IIV for *V_p2_*/*F* (sh_ηp) (%)	35.4 (60.4)	18.2	35.1	26.2–41.1
% CV for IIV for *K_a_* (sh_ηp) (%)	123.7 (33.9)	6.9	123.4	115.4–132.5
Covariance between CL/*F* and *V_c_*/*F*	0.209	4.7	0.209	0.190–0.228
% CV for proportional residual error (sh_ε) (%)	20.2 (17.8)	2.1	20.2	19.4–21.0

a CI, confidence interval; CV, coefficient of variation; IIV, interindividual variability; RSE, relative standard error; sh, shrinkage. CL/*F =* 10.8 × (body weight/67.7)^0.362^ × 0.519^Asian^; *Q*_1_/*F* = 12.4 × (body weight/67.7)^0.362^; *Q*_2_/*F* = 1.43 × (body weight/67.7)^0.362^; *V_c_*/F = 565 × (body weight/67.7)^0.833^ × 0.564^Asian^; *V_p1_*/*F* = 141 × (body weight/67.7)^0.833^; *V_p2_*/*F *= 139 × (body weight/67.7)^0.833^; *K_a_* (/h) = 1.03 × 0.682^gender^. Asian = 0 for non-Asian subject and 1 for Asian subject. Gender = 0 for male and 1 for female.

**FIG 1:**
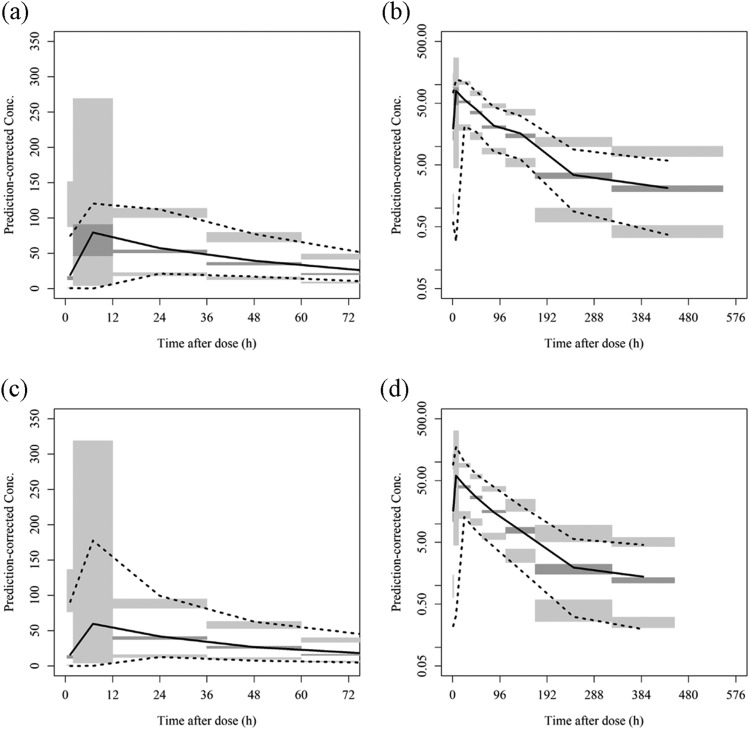
Prediction-corrected visual predictive check. Observed median (solid line) and 2.5th and 97.5th percentiles (dotted lines) of plasma concentrations were compared with their 95% prediction intervals (gray areas) simulated based on the final model. The figures are for phase 2/3 Asian patients in linear (a) and semilogarithmic (b) axes and for phase 2/3 non-Asian patients in linear (c) and semilogarithmic (d) axes.

Diagnostic plots for the final model are shown in Fig. S4. The final model adequately described the plasma concentrations, showing a good fit to the observed data and the absence of obvious bias. As shown in Fig. 1 and Fig. S5, the prediction-corrected visual predictive check (pc-VPC) (5) indicated that the model well captured the central trend of the observed data with a lack of bias, as the observed plasma concentration data and the median profiles and 95% predicted intervals (PIs) of the model were consistent. The stability and robustness of the final model were evaluated using the nonparametric bootstrap procedure (6) (Table 2). The parameter estimates in the final model had little bias and the final model was fairly robust, as the population parameter estimates obtained from 5,000 bootstrap sample sets were comparable to the estimates in the final model and the 80.1% of runs successfully completed.

### Pharmacokinetics in patients with risk factors of influenza complications.

The individual exposure indices of baloxavir acid (maximum plasma concentration [*C*_max_], area under the plasma concentration-time curve [AUC], and observed plasma concentration at 24 h after administration [*C*_24_]) were summarized by dose (body weight group) and race for the phase 3 OwH and HR studies (Table 3). All exposure parameters were lower in non-Asians than in Asians. The exposures in the phase 3 HR study were similar to those in the phase 3 OwH study regardless of dose or race. Relationships of ηs with risk factors in the final model are shown in Fig. S6. None of the risk factors evaluated in this study showed clear tendencies regarding η_CL/_*_F_*, suggesting that the pharmacokinetics of baloxavir acid would not be affected by the risk factors of influenza complications.

**TABLE 3:** Summary statistics for the predicted *C*_max_, predicted AUC, and observed *C*_24_ for influenza patients in phase 3 studies

Study	Dose	Race	No. of subjects (no. for *C*_24_)	*C*_max_ (ng/ml)^*a*^^,^^*b*^	AUC (ng·h/ml)^*a*^^,^^*b*^	*C*_24_ (ng/ml)^*a*^^,^^*c*^
Phase 3 otherwise healthy patients	40 mg (<80 kg)	Asian	309 (194)	97.6 (20.1–221)	6,210 (1,399–13,200)	59.8 (5.81–158)
		Non-Asian	59 (39)	63.9 (11.1–133)	3,648 (909.5–7,609)	37.2 (7.35–81.4)
	80 mg (≥80 kg)	Asian	34 (26)	136 (30.9–253)	9,741 (4,527–16,340)	74.9 (17.5–209)
		Non-Asian	44 (30)	94.6 (27.1–196)	6,345 (2,247–15,040)	88.7 (39.3–142)
	Overall patients		446 (289)	95.8 (11.1–253)	6,154 (909.5–16,340)	59.7 (5.81–209)
Phase 3 patients at high risk of influenza complications	40 mg (<80 kg)	Asian	138 (79)	104 (24.0–382)	6,380 (2,294–14,690)	64.0 (25.4–231)
		Non-Asian	96 (58)	60.6 (12.2–158)	3,661 (720.3–8,571)	35.9 (5.77–90.2)
	80 mg (≥80 kg)	Asian	26 (14)	137 (40.9–241)	9,733 (4,893–16,640)	87.6 (33.8–126)
		Non-Asian	118 (81)	84.9 (9.21–240)	5,737 (890.8–14,810)	58.7 (5.86–198)
	Overall patients		378 (232)	89.1 (9.21–382)	5,719 (720.3–16,640)	56.5 (5.77–231)

a Mean (range).

b *Post hoc* Bayesian estimation based on the population pharmacokinetic model.

c Observed plasma concentration at 20 to 28 h postdose.

### Exposure-response analysis.

**FIG 2:**
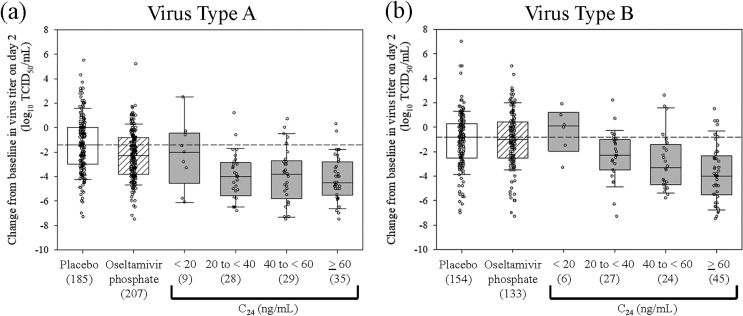
Relationships of change from baseline in virus titer on day 2 with *C*_24_ by virus type in patients at high risk of influenza complications. (a) Virus type A; (b) virus type B. The thick center line represents the median, the top and bottom of the box represent the 1st and 3rd quartiles, and whiskers represent the 10th and 90th percentiles. The dashed line represents the median value for placebo treatment. The number in parentheses is the number of subjects in each category.

The summary statistics of time to improvement of influenza symptoms (TTIIS; the primary endpoint) and the change from baseline in virus titer on day 2 (the secondary endpoint) by groups of exposure indices (*C*_max_, AUC, and *C*_24_) and by virus type are presented in Tables 4 and 5, respectively. Box plots of virus titer for *C*_24_ are shown in Fig. 2. For the three highest exposure categories of *C*_24_ at ≥20 ng/ml, baloxavir shortened the TTIIS compared to that of the placebo and, overall, was similar to oseltamivir for virus type A. For virus type B, for the two highest exposure categories, baloxavir shorteed the TTIIS compared to those of both placebo and oseltamivir, although TTIIS was highly variable in all groups and did not show clear exposure dependency. Other exposure indices show similar tendencies.

**TABLE 4:** Time to improvement of influenza symptoms by predicted *C*_max_, predicted AUC, and observed *C*_24_ groups

Parameter or other treatment	Range of pharmacokinetic parameter	Virus type A				Virus type B			
		*n*	Median (h)	Difference from placebo treatment (h)	Difference from oseltamivir phosphate treatment (h)	*n*	Median (h)	Difference from placebo treatment (h)	Difference from oseltamivir phosphate treatment (h)
*C*_max_ (ng/ml)	<40	38	101.5	0.4	35.0	21	93.5	0.3	−4.5
	40 to <80	70	75.6	−25.5	9.1	52	75.1	−18.1	−22.9
	80 to <120	56	67.0	−34.1	0.5	46	73.5	−19.7	−24.5
	≥120	44	50.3	−50.8	−16.2	45	67.3	−25.9	−30.7
AUC (ng·h/ml)	<2,500	24	101.1	0.0	34.6	11	85.3	−7.9	−12.7
	2,500 to <5,000	79	88.7	−12.4	22.2	58	84.6	−8.6	−13.4
	5,000 to <7,500	64	54.5	−46.6	−12.0	54	69.8	−23.4	−28.2
	≥7,500	41	59.5	−41.6	−7.0	41	68.4	−24.8	−29.6
*C*_24_ (ng/ml)	<20	11	165.2	64.1	98.7	6	89.0	−4.2	−9.0
	20 to <40	33	77.0	−24.1	10.5	30	90.7	−2.5	−7.3
	40 to <60	32	92.7	−8.4	26.2	25	68.6	−24.6	−29.4
	≥60	44	60.3	−40.8	−6.2	48	67.9	−25.3	−30.1
Placebo		214	101.1		34.6	167	93.2		−4.8
Oseltamivir phosphate		236	66.5	−34.6		148	98.0	4.8	

**TABLE 5:** Change from baseline in virus titer on day 2 by predicted *C*_max_, predicted AUC, and observed *C*_24_ groups

Parameter or other treatment	Range of pharmacokinetic parameter	Virus type A				Virus type B			
		*n*	Median (log_10_ [TCID_50_/ml])	Difference from placebo treatment (log_10_ [TCID_50_/ml])	Difference from oseltamivir phosphate treatment (log_10_ [TCID_50_/ml])	*n*	Median (log_10_ [TCID_50_/ml])	Difference from placebo treatment (log_10_ [TCID_50_/ml])	Difference from oseltamivir phosphate treatment (log_10_ [TCID_50_/ml])
*C*_max_ (ng/ml)	<40	31	−3.0	−1.6	−0.7	20	−1.1	−0.3	−0.1
	40 to <80	58	−3.8	−2.4	−1.5	47	−2.3	−1.5	−1.3
	80 to <120	48	−4.4	−3.0	−2.1	44	−2.7	−1.9	−1.7
	≥120	39	−4.2	−2.8	−1.9	38	−4.7	−3.9	−3.7
AUC (ng·h/ml)	<2,500	20	−3.0	−1.6	−0.7	9	−0.8	0.0	0.2
	2,500 to <5,000	64	−3.8	−2.4	−1.5	52	−2.3	−1.5	−1.3
	5,000 to <7,500	54	−3.9	−2.5	−1.6	52	−2.65	−1.85	−1.65
	≥7,500	38	−4.9	−3.5	−2.6	36	−3.9	−3.1	−2.9
*C*_24_ (ng/ml)	<20	9	−2.0	−0.6	0.3	6	0.1	0.9	1.1
	20 to <40	28	−4.0	−2.6	−1.7	27	−2.3	−1.5	−1.3
	40 to <60	29	−3.8	−2.4	−1.5	24	−3.3	−2.5	−2.3
	≥60	35	−4.5	−3.1	−2.2	45	−4.0	−3.2	−3.0
Placebo		185	−1.4		0.9	154	−0.8		0.2
Oseltamivir phosphate		207	−2.3	−0.9		133	−1.0	−0.2	

For type A virus, the change from baseline in virus titer on day 2 was more obvious in *C*_24_ groups of ≥20 ng/ml, with a median change of −3.8 to −4.5 log_10_(TCID_50_/ml) compared with −1.4 log_10_(TCID_50_/ml) for the placebo treatment group and −2.3 log_10_(TCID_50_/ml) for the oseltamivir treatment group. For type B virus, the change from baseline in virus titer on day 2 showed exposure-dependent declines, with a median change of −2.3 to −4.0 log_10_(TCID_50_/ml) for *C*_24_ groups of ≥20 ng/ml, which were larger reductions compared with those in the placebo treatment group (−0.8 log_10_[TCID_50_/ml]) and the oseltamivir treatment group (−1.0 log_10_[TCID_50_/ml]). In the lowest-exposure group, *C*_24_ < 20 ng/ml, less obvious reductions in TTIIS or virus titer were found for both type A and type B viruses. However, since the numbers of subjects in this group were limited, it is difficult to discuss the magnitude of the responses related to exposure levels.

## DISCUSSION

A population pharmacokinetic model of baloxavir acid was developed using data from 13 clinical studies including healthy subjects as well as patients who were either otherwise healthy or at high risk of influenza complications. Over the entire dose range explored, plasma drug exposure of baloxavir acid was well described by a three-compartment model with first-order elimination and absorption, indicating linear pharmacokinetics. All fixed and random effect parameters were precisely estimated, with relative standard error (RSE) below 20%. The η shrinkages for both CL/*F* and *V_c_*/*F* were low (4.4% and 10.9%, respectively), indicating that the sampling scheme was informative enough to obtain reliable empirical Bayesian estimates, while the η shrinkage for *V_p1_*/*F* and *V_p2_*/*F* was large, above 60%, indicating that the pharmacokinetic sampling scheme was much less informative for estimating the between-subject variability of disposition phase, which is not unexpected with a sparse sampling scheme.

Race and body weight were found to be relevant covariates of CL/*F* of baloxavir acid, which was consistent with findings from the previous population pharmacokinetic analysis (1). In both Asian and non-Asian patients, CL/*F* and *V_c_*/*F* of baloxavir acid increased with increasing body weight in a less-than-proportional manner, as shown by their allometric exponents estimated at 0.362 for CL/*F* and 0.833 for *V_c_*/*F*, which were both lower than the classical/theoretical ones of 0.75 and 1 (7, 8), respectively. Interestingly, an attempt to optimize body weight effect on both *V_c_*/*F* and CL/*F* for each race separately did not relevantly improve model fit (model no. 213 and 214 [Table S2c]), indicating that body weight effect on drug disposition may by itself not be ethnicity sensitive. *In vitro* data have shown that metabolic clearance of baloxavir involves primarily UGT1A3- and CYP3A-mediated metabolism, which is not known to be ethnicity sensitive. In addition, as excretion of unchanged baloxavir marboxil in urine or feces is primarily passive, neither is likely to be ethnicity sensitive. Moreover, the race effects on CL/*F* and *V_c_*/*F* were fairly similar, suggesting that the ethnic difference in drug disposition may represent a shift in oral bioavailability via *F* rather than a difference in systemic clearance (CL), although some minor ethnic differences in CL cannot be fully ruled out. The intrinsic and/or extrinsic factor driving ethnic difference in bioavailability is currently not known, but it might involve an interplay between prodrug conversion and the rate of downstream intestinal metabolism of baloxavir acid.

A gender effect was detected on the rate of absorption, with a 31.8% lower rate constant in females. Gender differences in gut transit times, lipid solubility of an agent, activities of certain CYP enzymes, diets, etc., have been reported to influence drug absorption and bioavailability (9). However, in the case of baloxavir, the gender difference in absorption may be multifactorial and difficult to elucidate, since the absorption process is likely to be a complex interplay that may involve biopharmaceutical factors (disintegration and dissolution in the gut), physiological factors (transit to absorption site), and bio- or physicochemical factors (food interaction, conversion from baloxavir marboxil to baloxavir acid by AADAC, and downstream intestinal metabolism or efflux). Since baloxavir acid has a wide therapeutic window with an apparent near plateau in response over the dose range explored, this finding is not deemed clinically relevant. No relevant differences in pharmacokinetics were found between otherwise healthy influenza patients and patients who are at high risk of influenza complications.

In the population pharmacokinetic analysis, a tendency of lower baloxavir acid exposure was seen when baloxavir marboxil was taken with food, in line with an observation made from a dedicated phase 1 food effect study (10). However, the magnitude of the overall food effect on *F* was small, and the covariate was eventually removed during refinement (backward deletion procedure) of the final model, since the estimates of the ratio on *F* approached 1 and the 95% CI was within the range of 0.80 to 1.25 (Fig. S1). This finding is in accordance with the previous population pharmacokinetic model in otherwise healthy influenza patients, in whom the effect of food intake on baloxavir acid exposure was found to be small (0.869-fold) and not clinically relevant (1). Indeed, considering its wide therapeutic window, the minor decrease in drug exposure when taken with food supports the label claim that baloxavir marboxil can be taken regardless of food intake (Xofluza package insert).

Body weight-based dosing used in both phase 3 OwH and HR studies (40 mg for 40 kg to <80 kg and 80 mg for ≥80 kg) successfully avoided underexposure in higher-body-weight individuals, regardless of race (Table 3). The lowest drug exposure was generally seen in non-Asian patients with body weights of <80 kg, in both OwH and HR patients. However, the drug exposure remained well above the mean exposure (*C*_max_, 27.8 ng/ml; AUC, 2,105 ng·h/ml; and *C*_24_, 15.1 ng/ml) observed at the lowest dose (10 mg) explored in the phase 2 OwH study (1, 11), which was demonstrated to be effective for both type A (statistically) and type B (numerically) influenza virus compared with the placebo treatment group (2). Thus, since baloxavir marboxil is safe and well tolerated up to 80 mg regardless of race and is efficacious over a wide exposure range; its therapeutic window appears to be wide and allows a globally aligned body weight-based dosing regimen that provides efficacious and safe exposure of baloxavir acid regardless of race or gender.

In the exposure-response analysis for influenza patients at high risk of complications, the median changes from the baseline in virus titer on day 2 were −3.8 to −4.5 log_10_(TCID_50_/ml) for type A virus and −2.3 to −4.0 log_10_(TCID_50_/ml) for type B virus for *C*_24_ exposure groups above or equal to 20 ng/ml, which were similar to those seen in the otherwise healthy influenza patients (−4.5 to −5.0 log_10_[TCID_50_/ml] for type A virus and −2.5 to −4.1 log_10_[TCID_50_/ml] for type B virus) reported in the previous paper (1). In the *C*_24_ exposure group of <20 ng/ml, the median changes from the baseline in virus titer on day 2 were −2.0 log_10_(TCID_50_/ml) for type A virus and 0.1 log_10_(TCID_50_/ml) for type B virus, which were smaller than those in OwH patients (−4.5 log_10_[TCID_50_/ml] for type A virus and −2.55 log_10_[TCID_50_/ml] for type B virus). However, the numbers of subjects were much smaller (9 for type A virus and 6 for type B virus) than those reported in the analysis with otherwise healthy influenza patients (66 for type A virus and 16 for type B virus) (1). This difference in the lowest *C*_24_ exposure group between OwH and HR patients is thought to be due to the small sample size in the phase 3 HR study, in which the effect of lower dose levels of 10 mg and 20 mg was not investigated. These results suggested that risk factors of influenza complications did not affect the virus reductions.

In conclusion, the population pharmacokinetics of baloxavir acid was successfully described by a three-compartment model with first-order elimination and first-order absorption with lag time based on the integrated data collected from healthy subjects, otherwise healthy patients, and patients at high risk of influenza complications. The model well described the plasma concentration data, and body weight and race were found to be the most important factors influencing clearance and volume of distribution. The exposures in high-risk patients were similar to those in otherwise healthy patients, and no pharmacokinetic difference was identified regarding any risk factors for influenza complications. The exposure-response analyses in high-risk patients showed that the body weight-based dose regimen (40 mg for the patients weighing less than 80 kg and 80 mg for the patients weighing at least 80 kg) shortened TTIIS and reduced virus titer for both type A and B influenza virus regardless of exposure levels to baloxavir acid of ≥20 ng/ml (*C*_24_). The population pharmacokinetic model and exposure-response analysis results for patients with risk factors of influenza complications are useful for understanding the pharmacokinetic and pharmacodynamic (PD) characteristics of baloxavir marboxil and also for optimization of dose regimens in clinical situations.

## MATERIALS AND METHODS

### Data for analysis.

Table S1 provides information of clinical studies used for the population pharmacokinetic analysis. All clinical studies were approved by the ethics committees for each site and conducted in compliance with the Declaration of Helsinki and good clinical practice (GCP). Baloxavir marboxil was administered orally in all these clinical studies. The details of study designs and dosages for the phase 2 OwH, phase 3 OwH, and phase 3 HR studies have been described in previous papers (2, 3). The patients in the phase 3 HR study were considered to be at high risk of influenza complications due to the presence of at least one of the inclusion criteria defined by the CDC.

Determination of plasma baloxavir acid concentrations was conducted using a validated liquid chromatography-tandem mass spectrometry method (more details in reference 1) at Sumika Chemical Analysis Service, Ltd. (Osaka, Japan), for phase 1 studies and a phase 2 study, or at LGC Limited (Teddington, UK) for two phase 3 studies.

Pharmacokinetic and pharmacodynamic data from the phase 3 HR study were used for the exposure-response analysis. The TTIIS and the change from baseline in the influenza virus titer on day 2 were used as efficacy parameters. The TTIIS was defined as the time from the start of study treatment to the improvement of influenza symptoms. The virus titers were determined, and cytopathic effects were evaluated by the methods described in reference 1.

### Population pharmacokinetic analysis.

Nonlinear mixed-effect modeling software, NONMEM (version 7.3; ICON Development Solutions, USA), was used for the population pharmacokinetic analysis with a PREDPP library and NM-TRAN preprocessor. A first-order conditional estimation with interaction was used for the analysis.

First, using 11,848 concentration data points from 1,827 subjects, a basic structural population pharmacokinetic model was constructed without any covariate. One-, two-, and three-compartment models with first-order elimination and first-order absorption with lag time were tested for the structural model. The interindividual variability (IIV) on pharmacokinetic parameters was assumed to follow a log-normal distribution. A model for residual error variability was selected from a proportional-error model, an additive-error model, and a combined-error model.

Next, a covariate model was constructed by combining the preliminary covariates screening through univariate regression analysis with a forward selection and a stepwise backward deletion. The tested covariates are summarized in Table 1 with pharmacokinetic parameters. The body surface area-adjusted estimated glomerular filtration rate (eGFR) and creatinine clearance (CL_CR_) were calculated by the methods described in reference 1. A power model for a continuous variable and a multiplicative model for a categorical variable were used for the analysis as described in reference 1. The significance level of 0.05 based on the χ^2^ test (*P* < 0.05; difference in objective function value [ΔOBJ] is less than −3.84 for 1 degree of freedom) was used for the screening and forward selection. The significant covariates at screening were tested in the forward selection to construct a full model. The significance level of 0.01 based on the χ^2^ test (*P* < 0.01; ΔOBJ is more than 6.63 for 1 degree of freedom) was used for the stepwise backward deletion to construct the final model.

The points with absolute conditional weighted residuals with interaction (CWRESI) value of ≥6 in the base model and the final model were excluded from the analysis as outlier concentrations.

### Model evaluation.

The population pharmacokinetic model was evaluated using diagnostic plots of the observed plasma concentration (DV) versus the mean population predicted plasma concentration (PRED), DV versus individual predicted plasma concentrations (IPRED), absolute individual weighted residuals (IWRES) versus IPRED, CWRESI versus PRED, and CWRESI versus time after the reference dose. A nonparametric bootstrap resampling procedure (6) was performed using Perl-speaks-NONMEM (version 4.2) to assess the stability of the final parameter estimates and the robustness of the final model. The 5,000 bootstrap sample sets were resampled from the original data set, and the parameter estimates for each of the 5,000 sample sets were obtained using the final model. The medians and 95% CIs of the 5,000 parameter estimates from bootstrap sample sets were compared with the mean and standard error of the final parameter for each parameter estimate.

A pc-VPC (5) was performed and the final model was evaluated by comparing the observed plasma concentrations with the 95% PIs simulated from the final population pharmacokinetic parameters. Five hundred data sets were simulated from the final population pharmacokinetic parameters using the original data set as a simulation template. For each simulation run, the median and the 2.5th and 97.5th percentiles of pharmacokinetic time course were first calculated. Next, for each of those statistics, the 95% CIs were computed over the 500 simulated runs and compared with the observations.

### Exposure-response analysis.

The individual systemic exposures of baloxavir acid, *C*_max_ and AUC, were calculated using the individual *post hoc* pharmacokinetic parameter estimates derived from the final model using an empirical Bayesian estimation.

Exposure-response analyses were performed for TTIIS on a total of 658 measurements and for the change from the baseline in the virus titer on day 2 on a total of 568 measurements from the phase 3 HR study. These estimated individual *C*_max_, individual AUC, and *C*_24_ (sampling time window: 20 to 28 h postdose) were used as exposure indices. The TTIIS and change from baseline in virus titer on day 2 were plotted against the exposure indices (Fig. S7 and S8). In addition, four groups of patients were created based on different exposure levels: *C*_max_ levels (<40 ng/ml, 40 to <80 ng/ml, 80 to <120 ng/ml, and ≥120 ng/ml), AUC levels (<2,500 ng·h/ml, 2,500 to <5,000 ng·h/ml, 5,000 to <7,500 ng·h/ml, and ≥7,500 ng·h/ml), or *C*_24_ levels (<20 ng/ml, 20 to <40 ng/ml, 40 to <60 ng/ml, and ≥60 ng/ml); the TTIIS and change from baseline in virus titer on day 2 were then summarized and graphically presented for comparison across the baloxavir acid exposure groups and with the oseltamivir and placebo treatment groups (Fig. 2 and Fig. S9 and S10). To facilitate the comparison with the previous exposure-response analyses for otherwise healthy influenza patients (1), the ranges of the exposure categories were kept the same.

## Supplementary Material

Supplemental file 1
