# Supplementary material for: Population Pharmacokinetics and Exposure-Response Relationships of Baloxavir Marboxil in Influenza Patients at High Risk of Complications
Source: Antimicrob Agents Chemother. 2020 Jun 23;64(7):e00119-20. doi: 10.1128/AAC.00119-20 (PMC7318003; doi:10.1128/AAC.00119-20)

Supplemental material

Table S1 Summary of the clinical studies used for population pharmacokinetic analysis

**Study Title**	**Country**	**Dosage/ Dosing Design**	**Population and Number of Subjects with PK data**	**Sampling Points/Subjects**	**Number of plasma concentration data used for PPK analysis**
Single Dose Study	JP	6 - 80 mg Single dose	Healthy male subjects N = 30	19 points/subject	528 points
BA/FE Study	JP	20 mg Single dose	Healthy male subjects N = 29	17 × 2 points/subject	668 points
DDI Study with Itraconazole	US	20 mg Single dose	Healthy subjects N = 12	18 points/subject[a]	203 points
Thorough QTc Study	JP	40, 80 mg Single dose	Healthy subjects N = 64	16 × 2 points/subject	1827 points
Mass Balance Study	UK	40 mg Single dose	Healthy male subjects N = 6	19 points/subject	108 points
DDI Study with Tamiflu®	JP	40 mg Single dose	Healthy male subjects N = 18	18 points/subject[a]	290 points
Hepatic Impairment Study	US	40 mg Single dose	Subjects with normal hepatic function N = 8 Subjects with moderate hepatic impairment N = 8	19 points/subject	285 points
DDI Study with Probenecid	US	80 mg Single	Healthy subjects N = 12	18 points/subject	204 points

		dose			
BE/FE study for 10-mg tablet	JP	20, 40 mg Single dose	Healthy male subjects N = 78	18 points/subject	1084
BE study for 2% Granule	JP	20 mg Single dose	Healthy male subjects N = 28	18 × 2 points/subject	958
Phase 2 Dose Finding Study in Otherwise Healthy Patients with Influenza	JP	10, 20, 40 mg Single dose	Otherwise healthy patients with influenza N = 300	2-9 points/subject	1338 points
Phase 3 DBT Study in Otherwise Healthy Patients with Influenza (CAPSTONE-1)	JP, US	40, 80 mg Single dose	Adult and adolescent otherwise healthy patients with influenza N = 586	2-5 points/subject	2014 points
Phase 3 DBT Study in Patients with Influenza at High Risk of Influenza Complications (CAPSTONE-2)	Global[b]	40, 80 mg Single dose	Adult and adolescent patients with influenza at high risk of influenza complications N = 664	2-5 points/subject	2341 points

BA, bioavailability; BE, bioequivalence; DBT, double blind treatment; DDI, drug-drug interaction; FE, food effect; JP, Japan; UK, United Kingdom; US, United States

[a] The concentrations for baloxavir marboxil treatment alone were used. The concentrations with perpetrators were excluded from the analyses for DDI studies.

[b] CAPSTONE-2 study was conducted in 16 countries in Asia, North America, Europe, South America, and Oceania.

Table S2 Model building process

(a) Base model

**Model No.**	**Model description**	**No. of subjects /DV points**	**OBJ**	**-2l.l.d. (ΔOBJ)**	**Nparm**	**Reference model**	**Significance/ Acceptance**	**Convergence**
001	1-compartment model with additive residual error	1827 / 11848	83602.724	-	7	-	NO	OK
002	1-compartment model with proportional residual error	1827 / 11848	69475.042	-14127.682	7	001	NO	OK
003	1-compartment model with combined residual error	1827 / 11848	66854.749	-2620.293	8	002	NO	OK
004	2-compartment model with additive residual error	1827 / 11848	80629.355	-	11	-	NO	OK
005	2-compartment model with proportional residual error	1827 / 11848	62781.916	-17847.439	11	004	NO	OK
006	2-compartment model with combined residual error	1827 / 11848	62746.402	-35.514	12	005	YES	OK
007	3-compartment model with additive residual error	1827 / 11848	-	-	15	-	NO	Minimization terminated
008	3-compartment model with proportional residual error	1827 / 11848	-	-	15	007	NO	Minimization terminated
009	3-compartment model with combined residual error	1827 / 11848	-	-	16	008	NO	Minimization terminated
010	Model No.006 with absorption lag time (ALAG1) without inter-individual variability for ALAG1	1827 / 11848	61489.790	-1256.612	13	006	YES	OK [a]
011	Remove inter-individual variability for Q1/F from Model No.010	1827 / 11848	61489.972	0.182	12	010	YES	OK
**011 (Base model)**	**Remove a point for which |CWRESI| was more than 6**	**1827 / 11847**	**61453.399**	**-**	**12**	**-**	**YES**	**OK**

Nparm = the number of estimable parameters; OBJ = objective function value; -2l.l.d. = minus twice the log-likelihood difference

[a] Parameter estimate was near its boundary.

(b) 1st screening for covariates

Model No.	Model description	No. of subjects /DV points	OBJ	-2l.l.d. (ΔOBJ)	Nparm	Reference model	Significance/ Acceptance	Convergence
101	Model No.011 with effect of body weight on CL/F	1827 / 11847	61004.594	-448.805	13	011	YES	OK
102	Model No.011 with effect of age on CL/F	1827 / 11847	61447.102	-6.297	13	011	YES	OK
103	Model No.011 with effect of BMI on CL/F	1827 / 11847	61053.010	-400.389	13	011	YES	OK
104	Model No.011 with effect of gender on CL/F	1827 / 11847	61453.133	-0.266	13	011	NO	OK
105	Model No.011 with effect of AST on CL/F	1827 / 11847	61449.227	-4.172	13	011	YES	OK
106	Model No.011 with effect of ALT on CL/F	1827 / 11847	61432.907	-20.492	13	011	YES	OK
107	Model No.011 with effect of eGFRadj on CL/F	1827 / 11847	61387.446	-65.953	13	011	YES	OK
108	Model No.011 with effect of eGFRabs on CL/F	1827 / 11847	61163.653	-289.746	13	011	YES	OK
109	Model No.011 with effect of CLcr on CL/F	1827 / 11847	61329.816	-123.583	13	011	YES	OK
110	Model No.011 with effect of race (Asian) on CL/F	1827 / 11847	60428.756	-1024.643	13	011	YES	OK
111	Model No.011 with effect of race (White) on CL/F	1827 / 11847	60876.634	-576.765	13	011	YES	OK
112	Model No.011 with effect of race (Black or African American) on CL/F	1827 / 11847	61323.052	-130.347	13	011	YES	OK
113	Model No.011 with effect of health status (healthy subjects) on CL/F	1827 / 11847	61355.474	-97.925	13	011	YES	OK
114	Model No.011 with effect of health status (patients with influenza) on CL/F	1827 / 11847	61382.378	-71.021	13	011	YES	OK
115	Model No.011 with effect of health status (patients without influenza) on CL/F	1827 / 11847	61031.039	-422.360	13	011	YES	OK
116	Model No.011 with effect of body weight on Vc/F	1827 / 11847	60817.272	-636.127	13	011	YES	OK
117	Model No.011 with effect of age on Vc/F	1827 / 11847	61444.994	-8.405	13	011	YES	OK
118	Model No.011 with effect of BMI on Vc/F	1827 / 11847	60995.408	-457.991	13	011	YES	OK

Model No.	Model description	No. of subjects /DV points	OBJ	-2l.l.d. (ΔOBJ)	Nparm	Reference model	Significance/ Acceptance	Convergence
119	Model No.011 with effect of gender on Vc/F	1827 / 11847	61443.831	-9.568	13	011	YES	OK
120	Model No.011 with effect of race (Asian) on Vc/F	1827 / 11847	61105.965	-347.434	13	011	YES	OK
121	Model No.011 with effect of race (White) on Vc/F	1827 / 11847	61123.528	-329.871	13	011	YES	OK
122	Model No.011 with effect of race (Black or African American) on Vc/F	1827 / 11847	61377.308	-76.091	13	011	YES	OK
123	Model No.011 with effect of health status (healthy subjects) on Vc/F	1827 / 11847	61393.265	-60.134	13	011	YES	OK
124	Model No.011 with effect of health status (patients with influenza) on Vc/F	1827 / 11847	61376.801	-76.598	13	011	YES	OK
125	Model No.011 with effect of health status (patients without influenza) on Vc/F	1827 / 11847	61144.756	-308.643	13	011	YES	OK
126	Model No.011 with effect of age on Ka	1827 / 11847	61452.374	-1.025	13	011	NO	OK
127	Model No.011 with effect of gender on Ka	1827 / 11847	61426.338	-27.061	13	011	YES	OK
128	Model No.011 with effect of food condition (fasted) on F	1827 / 11847	61448.361	-5.038	13	011	YES	OK
129	Model No.011 with effect of food condition (fed) on F	1827 / 11847	61436.841	-16.558	13	011	YES	OK
130	Model No.011 with effect of food condition (intermediate) on F	1827 / 11847	61426.863	-26.536	13	011	YES	OK
131	Model No.011 with effect of race (Asian) on F	1827 / 11847	60004.854	-1448.545	13	011	YES	OK
132	Model No.011 with effect of race (White) on F	1827 / 11847	60518.917	-934.482	13	011	YES	OK
133	Model No.011 with effect of race (Black or African American) on F	1827 / 11847	61236.408	-216.991	13	011	YES	OK
134	Model No.011 with effect of risk factor (Asthma or chronic lung disease) on CL/F	1827 / 11847	61313.982	-139.417	13	011	YES	OK
135	Model No.011 with effect of risk factor (Endocrine disorders) on CL/F	1827 / 11847	61405.738	-47.661	13	011	YES	OK

Model No.	Model description	No. of subjects /DV points	OBJ	-2l.l.d. (ΔOBJ)	Nparm	Reference model	Significance/ Acceptance	Convergence
136	Model No.011 with effect of risk factor (Neurological and neurodevelopmental disorders) on CL/F	1827 / 11847	61427.728	-25.671	13	011	YES	OK
137	Model No.011 with effect of risk factor (Heart disease) on CL/F	1827 / 11847	61427.972	-25.427	13	011	YES	OK
138	Model No.011 with effect of risk factor (Adults ≥ 65 years of age) on CL/F	1827 / 11847	61447.822	-5.577	13	011	YES	OK
139	Model No.011 with effect of risk factor (Metabolic disorders) on CL/F	1827 / 11847	61453.194	-0.205	13	011	NO	OK
140	Model No.011 with effect of risk factor (Morbid obesity) on CL/F	1827 / 11847	61378.913	-74.486	13	011	YES	OK

Nparm = the number of estimable parameters; OBJ = objective function value; -2l.l.d. = minus twice the log-likelihood difference

The risk factors with subpopulation under 5% of the whole population in the phase 3 HR study (residents of long-term care facilities, compromised immune system, American Indian and Alaska Natives, blood disorders, women who are within 2 weeks postpartum and are not breastfeeding) were not tested.

(c) Covariate model for body weight and race

Model No.	Model description	No. of subjects /DV points	OBJ	-2l.l.d. (ΔOBJ)	Nparm	Reference model	Significance/ Acceptance	Convergence
110	Model No.011 with effect of race (Asian) on CL/F	1827 / 11847	60428.756	-	13	-	-	-
201[a]	Model No.110 with effect of body weight on Vc/F	1827 / 11847	59754.702	-674.054	14	110	YES	OK
202[a]	Model No.201 with effect of body weight on CL/F	1827 / 11847	59627.583	-127.119	15	201	YES	OK
203[a]	Model No.202 with effect of race (Asian) on Vc/F	1827 / 11847	59490.416	-137.167	16	202	YES	OK
206	3-compartment model with combined residual error based on Model No.203, without inter-individual variability for Q2/F and Vp2/F	1827 / 11847	58891.931	-598.485	18	203	YES	OK
207	Model No.206 with inter-individual variability for Vp2/F	1827 / 11847	58882.578	-9.353	19	206	YES	OK
208	Model No.207 with inter-individual variability for Q1/F	1827 / 11847	58882.578	0.000	20	207	NO	OK
209	Model No.207 with inter-individual variability for Q2/F	1827 / 11847	58882.578	0.000	20	207	NO	OK
210	Model No.207 with proportional residual error	1827 / 11847	58883.253	0.675	18	207	YES	OK
211	Model No.210 with effect of body weight on Vp1/F and Vp2/F (the same θ with Vc/F)	1827 / 11847	58756.069	-127.184	18	210	YES	OK
**212 (Body weight and race model)**	**Model No.211 with effect of body weight on Q1/F and Q2/F (the same θ with CL/F)**	**1827 / 11847**	**58729.585**	**-26.484**	**18**	**211**	**YES**	**OK**
213	Model No.212 with estimating the effect of body weight on CL/F, Q1/F, and Q2/F (the same θ for exponent) by race (Asian and non-Asian)	1827 / 11847	58715.364	-14.221	19	212	NO[b]	OK

Model No.	Model description	No. of subjects /DV points	OBJ	-2l.l.d. (ΔOBJ)	Nparm	Reference model	Significance/ Acceptance	Convergence
214	Model No.212 with estimating the effect of body weight on Vc/F, Vp1/F, and Vp2/F (the same θ for exponent) by race (Asian and non-Asian)	1827 / 11847	58722.763	-6.822	19	212	NO[b]	OK

Nparm = the number of estimable parameters; OBJ = objective function value; -2l.l.d. = minus twice the log-likelihood difference

[a] As in the previous population pharmacokinetic analysis (3), the results from univariate regression analysis and plots of Bayesian-estimated θs and ηs versus covariates for the base model (Supplementary Figure S3) indicated that the effect of body weight and race were the most important covariates, then, the effects of body weight and race on the pharmacokinetic parameters (CL/F and Vc/F) were investigated first.

[b] The different effects of body weight by race were not accepted since the improvement of the model based on ΔOBJ was small and the estimated effects of body weight were not different from each other relevantly.

(d) 2nd screening for covariates

Model No.	Model description	No. of subjects /DV points	OBJ	-2l.l.d. (ΔOBJ)	Nparm	Reference model	Significance/ Acceptance	Convergence
301	Model No.212 with effect of age on CL/F	1827 / 11847	58724.871	-4.714	19	212	YES	OK
302	Model No.212 with effect of gender on CL/F	1827 / 11847	58745.680	16.095	19	212	NO	OK
303	Model No.212 with effect of AST on CL/F	1827 / 11847	58750.293	20.708	19	212	NO	OK
304	Model No.212 with effect of ALT on CL/F	1827 / 11847	58699.401	-30.184	19	212	YES	OK
311	Model No.212 with effect of age on Vc/F	1827 / 11847	58720.967	-8.618	19	212	YES	OK
312	Model No.212 with effect of gender on Vc/F	1827 / 11847	58705.074	-24.511	19	212	YES	OK
316	Model No.212 with effect of age on Ka	1827 / 11847	58757.946	28.361	19	212	NO	OK
317	Model No.212 with effect of gender on Ka	1827 / 11847	58702.032	-27.553	19	212	YES	OK
318	Model No.212 with effect of food condition (fasted) on F	1827 / 11847	58690.295	-39.290	19	212	YES	OK
319	Model No.212 with effect of food condition (intermediate) on F	1827 / 11847	58749.952	20.367	19	212	NO	OK
320	Model No.212 with effect of food condition (fed) on F	1827 / 11847	58679.623	-49.962	19	212	YES	OK
321	Model No.212 with effect of risk factor (Asthma or chronic lung disease) on CL/F	1827 / 11847	58744.128	14.543	19	212	NO	OK
322	Model No.212 with effect of risk factor (Endocrine disorders) on CL/F	1827 / 11847	58729.902	0.317	19	212	NO	OK
323	Model No.212 with effect of risk factor (Neurological and neurodevelopmental disorders) on CL/F	1827 / 11847	58548.820	-180.765	19	212	YES/Not tested [a]	OK
324	Model No.212 with effect of risk factor (Heart disease) on CL/F	1827 / 11847	58554.716	-174.869	19	212	YES/Not tested [a]	OK
325	Model No.212 with effect of risk factor (Adults >= 65 years of age) on CL/F	1827 / 11847	58714.911	-14.674	19	212	YES/Not tested [a]	OK

Model No.	Model description	No. of subjects /DV points	OBJ	-2l.l.d. (ΔOBJ)	Nparm	Reference model	Significance/ Acceptance	Convergence
326	Model No.212 with effect of risk factor (Metabolic disorders) on CL/F	1827 / 11847	58736.911	7.326	19	212	NO	OK
327	Model No.212 with effect of risk factor (Morbid obesity) on CL/F	1827 / 11847	58727.786	-1.799	19	212	NO	OK

Nparm = the number of estimable parameters; OBJ = objective function value; -2l.l.d. = minus twice the log-likelihood difference

[a] The effect was not tested in the forward selection procedure because it seemed no effect visually.

Univariate regression analysis for BMI was not performed since BMI was highly correlated with body weight, and the effect of body weight had been already included in the model.

Univariate regression analysis for health status was not performed since the distribution of race versus health status were unbalanced and it was difficult to distinguish each effect.

Univariate regression analysis for eGFRadj, eGFRabs, or CLcr was not performed since there were not clear relationships between $\eta_{CL/F}$ in Model No.212 and eGFRadj, eGFRabs, and CLcr visually, and elimination of baloxavir acid via kidney is considered to be minor.

(e) Full model

Model No.	Model description	No. of subjects /DV points	OBJ	-2l.l.d. (ΔOBJ)	Nparm	Reference model	Significance/ Acceptance	Convergence
320	Model No.212 with effect of food condition (fed) on F	1827 / 11847	58679.623	-	19	-	-	-
401	Model No.320 with effect of food condition (fasted) on F	1827 / 11847	58680.430	0.807	20	320	NO	OK
402	Model No.320 with effect of ALT on CL/F	1827 / 11847	58667.981	-11.642	20	320	YES	OK
403	Model No.402 with effect of gender on Ka	1827 / 11847	58601.300	-66.681	21	402	YES	OK
404	Model No.403 with effect of gender on Vc/F	1827 / 11847	58546.907	-54.393	22	403	YES	OK
405	Model No.404 with effect of age on Vc/F	1827 / 11847	58520.488	-26.419	23	404	YES	OK
406	Model No.405 with effect of age on CL/F	1827 / 11847	58499.336	-21.152	24	405	YES	OK
**407 (Full model)**	**Model No.406 with covariance between CL/F and Vc/F**	**1827 / 11847**	**57434.197**	**-1065.139**	**25**	**406**	**YES**	**OK**

Nparm = the number of estimable parameters; OBJ = objective function value; -2l.l.d. = minus twice the log-likelihood difference

(f) Final model

Model No.	Model description	No. of subjects /DV points	OBJ	-2l.l.d. (ΔOBJ)	Nparm	Reference model	Significance/ Acceptance	Convergence
407	Model No.406 with covariance between CL/F and Vc/F	1827 / 11847	57434.197	-	25	-	-	-
507	Model No.407 without effect of BWT on CL/F, Q1/F and Q2/F	1827 / 11847	-	-	24	407	NO	Minimization terminated
514	Model No.407 without effect of BWT on Vc/F, Vp1/F and Vp2/F	1827 / 11847	57812.541	378.344	24	407	NO	OK
515	Model No.407 without effect of race (Asian) on CL/F	1827 / 11847	58121.676	687.479	24	407	NO	OK
516	Model No.407 without effect of race (Asian) on Vc/F	1827 / 11847	57669.778	235.581	24	407	NO	OK
517	Model No.407 without effect of food (fed) on F	1827 / 11847	57484.416	50.219	24	407	NO	OK
518	Model No.407 without effect of ALT on CL/F	1827 / 11847	57459.605	25.408	24	407	NO	OK
519	Model No.407 without effect of gender on Ka	1827 / 11847	57456.892	22.695	24	407	NO	OK
520	Model No.407 without effect of gender on Vc/F	1827 / 11847	57440.880	6.683	24	407	NO	OK
521	Model No.407 without effect of age on Vc/F	1827 / 11847	57450.958	16.761	24	407	NO	OK
522	Model No.407 without effect of age on CL/F	1827 / 11847	57447.077	12.880	24	407	NO	OK
523	Model No.407 without effect of ALT on CL/F, age on CL/F, age on Vc/F, gender on Vc/F, and food (fed) on F	1827 / 11847	57532.479	98.282	20	407	YES	OK
**523 (Final model)**	**Remove a point for which |CWRESI| was more than 6**	**1827 / 11846**	**57507.712**	**-**	**20**	**-**	**YES**	**OK**
524	Model No 523 without ALAG1 and with one-transit compartment absorption model with inter-individual variability for transit time (Ktr)	1827/11846	-	-	21	523	NO	Rounding error[a]
525	Model No 524 with two-transit compartment absorption model	1827/11846	-	-	21	524	NO	Rounding

Model No.	Model description	No. of subjects /DV points	OBJ	-2l.l.d. (ΔOBJ)	Nparm	Reference model	Significance/ Acceptance	Convergence
								error[a]

Nparm = the number of estimable parameters; OBJ = objective function value; -2l.l.d. = minus twice the log-likelihood difference

[a] The runs were unstable with frequent rounding error.

Table S3 Population pharmacokinetic parameter estimates in the base model

**Parameter**	**Units**	**Base model**	
		**Estimate**	**%RSE**
Pharmacokinetic parameters			
CL/F	L/h	7.27	1.4
Vc/F	L	467	2.1
Q1/F	L/h	5.14	3.8
Vp1/F	L	207	1.8
Ka	1/h	1.18	4.9
Lag time	h	0.364	2.9
% CV for IIV for CL/F (sh_ηp)	%	53.5 (4.2)	4.0
% CV for IIV for Vc/F (sh_ηp)	%	69.4 (10.8)	4.6
% CV for IIV for Vp1/F (sh_ηp)	%	24.7 (50.7)	10.5
% CV for IIV for Ka (sh_ηp)	%	130.0 (34.1)	6.0
% CV for proportional residual error (sh_ε)	%	20.9 (18.7)	2.1
additive residual error	ng/mL	0.0710	31.7

CV, coefficient of variation; IIV, inter-individual variability, RSE, relative standard error; sh, shrinkage.

Figure S1 The effect magnitude of each covariate in (a) the full model and (b) the final model. The ratios of parameters of interest with certain values of covariate relative to the reference value and their 95% confidence intervals were estimated. Reference values: 67.7 kg for body weight, 38 years old for age, 18 U/L for ALT, non-Asian for race, male for gender, and fasted for food condition.

(a) Full model

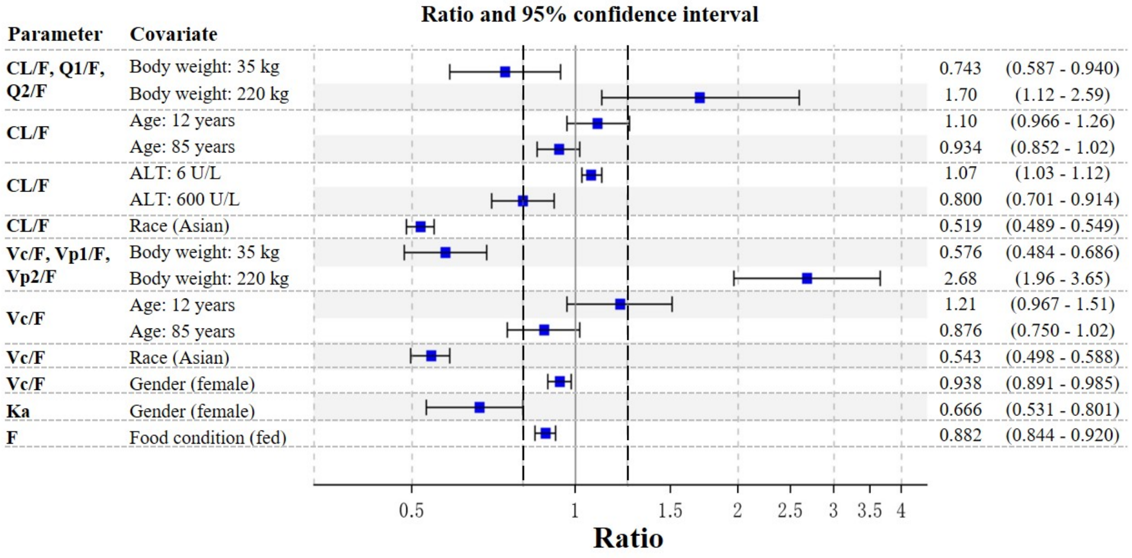

(b) Final Model

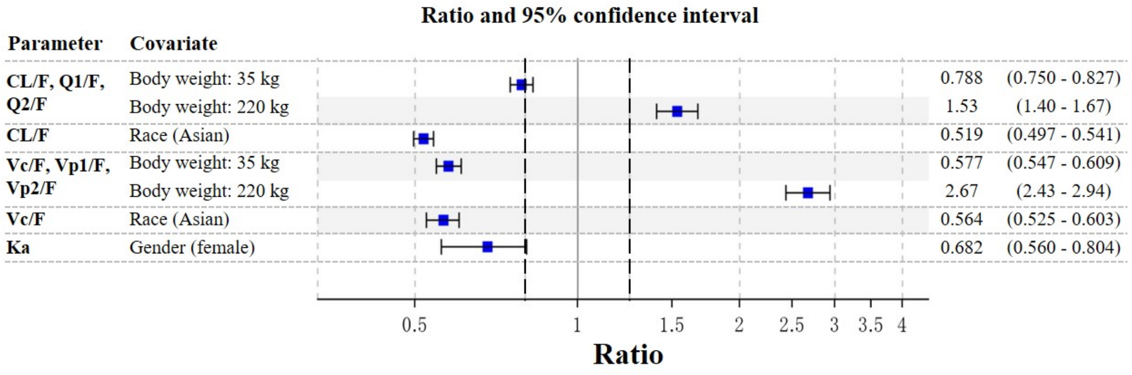

Figure S2 Scatter plots of individual η for (a) CL/F versus body weight, (b) Vc/F versus body weight, and boxplots of η for (d) CL/F by race (Asian versus non-Asian), (e) Vc/F by race (Asian versus non-Asian), and (f) Ka versus gender for the final model. Red dashed line in scatter plots represents a locally-weighted scatterplot smoothing line. Thick center line in box plots represents median, top and base of the box represent IQR, whiskers represent the most extreme data within 1.5 × IQR, and circles represent outliers beyond 1.5 × IQR.

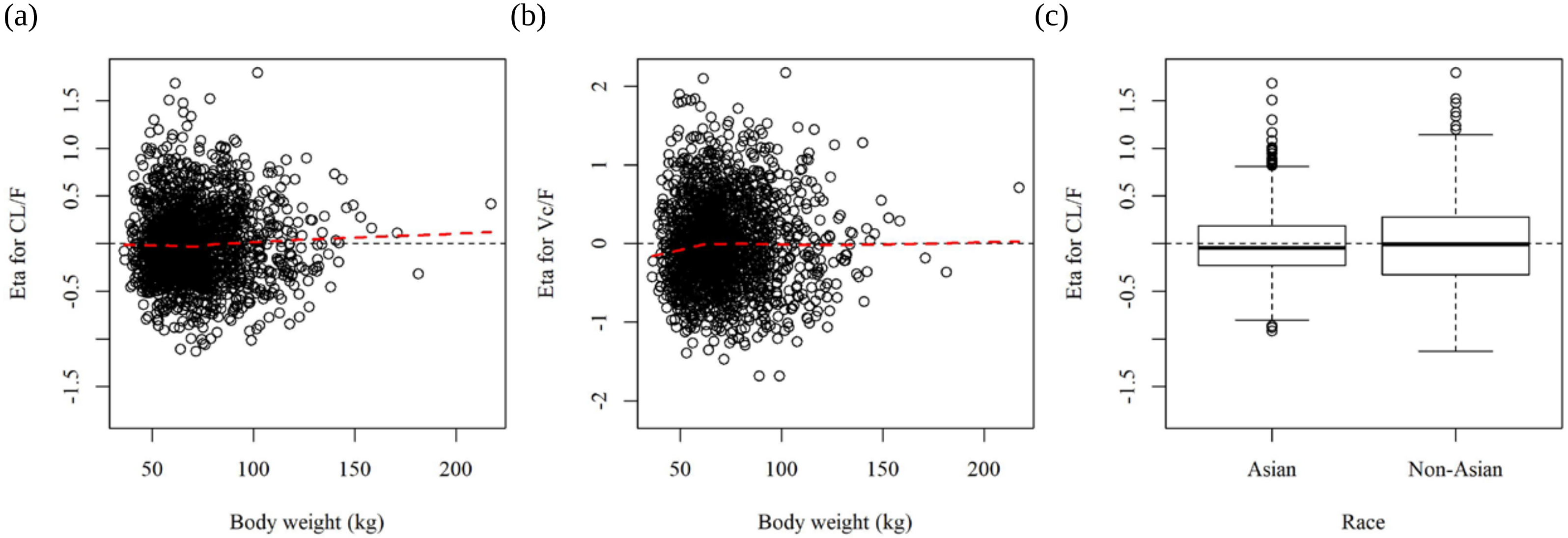

(d)

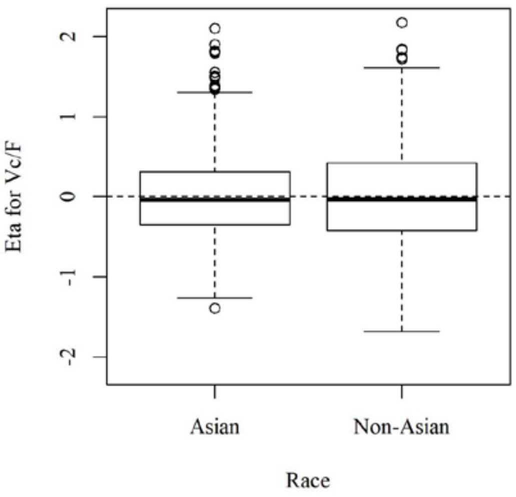

Eta for Vc/F
2
1
0
-1
-2
Asian
Non-Asian
Race

(e)

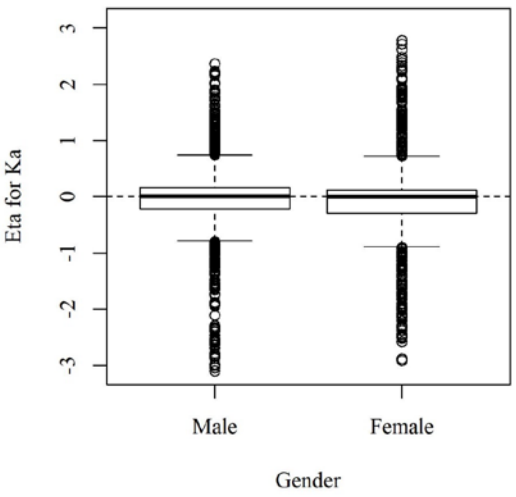

Eta for Ka
3
2
1
0
-1
-2
-3
Male
Female
Gender

Figure S3 Relationships between body weight and (a) individual CL/F, (b) individual Vc/F, (c) individual $\eta_{CL/F}$, and (d) individual $\eta_{Vc/F}$, boxplots for (e) CL/F, (f) Vc/F, (g) $\eta_{CL/F}$, and (h) $\eta_{Vc/F}$ by Asian or non-Asian. Individual CL/F and Vc/F values were estimated by an empirical Bayesian method based on the base model. Red dashed line represents a locally-weighted scatterplot smoothing line. Thick center line represents median, top and base of the box represent the first and third quartiles [interquartile range (IQR)], whiskers represent the most extreme data within 1.5 × IQR, and circles represent outliers beyond 1.5 × IQR.

(a)

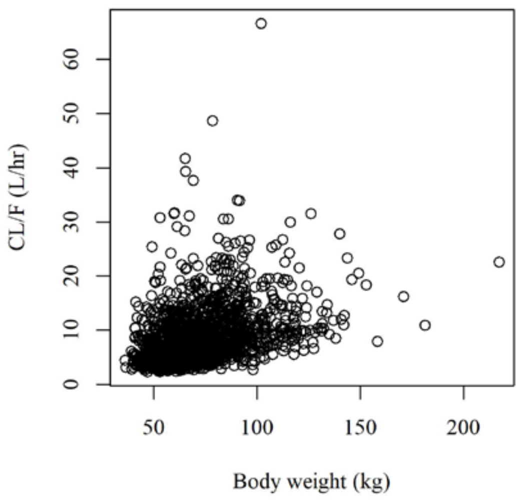

(b)

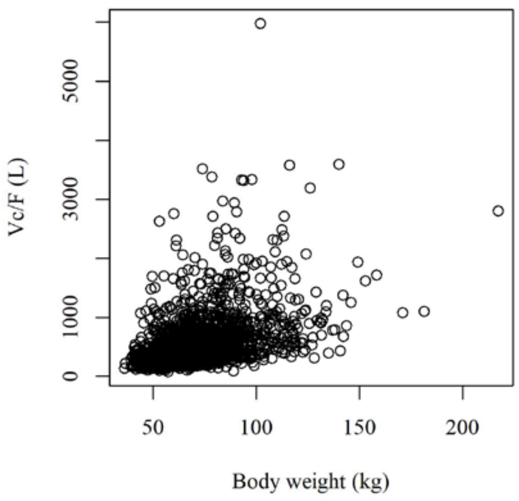

(c)

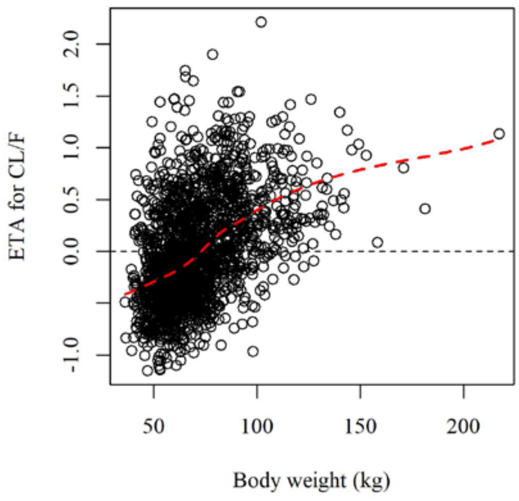

(d)

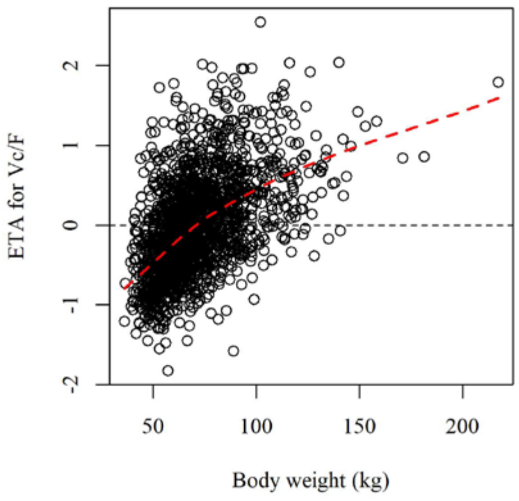

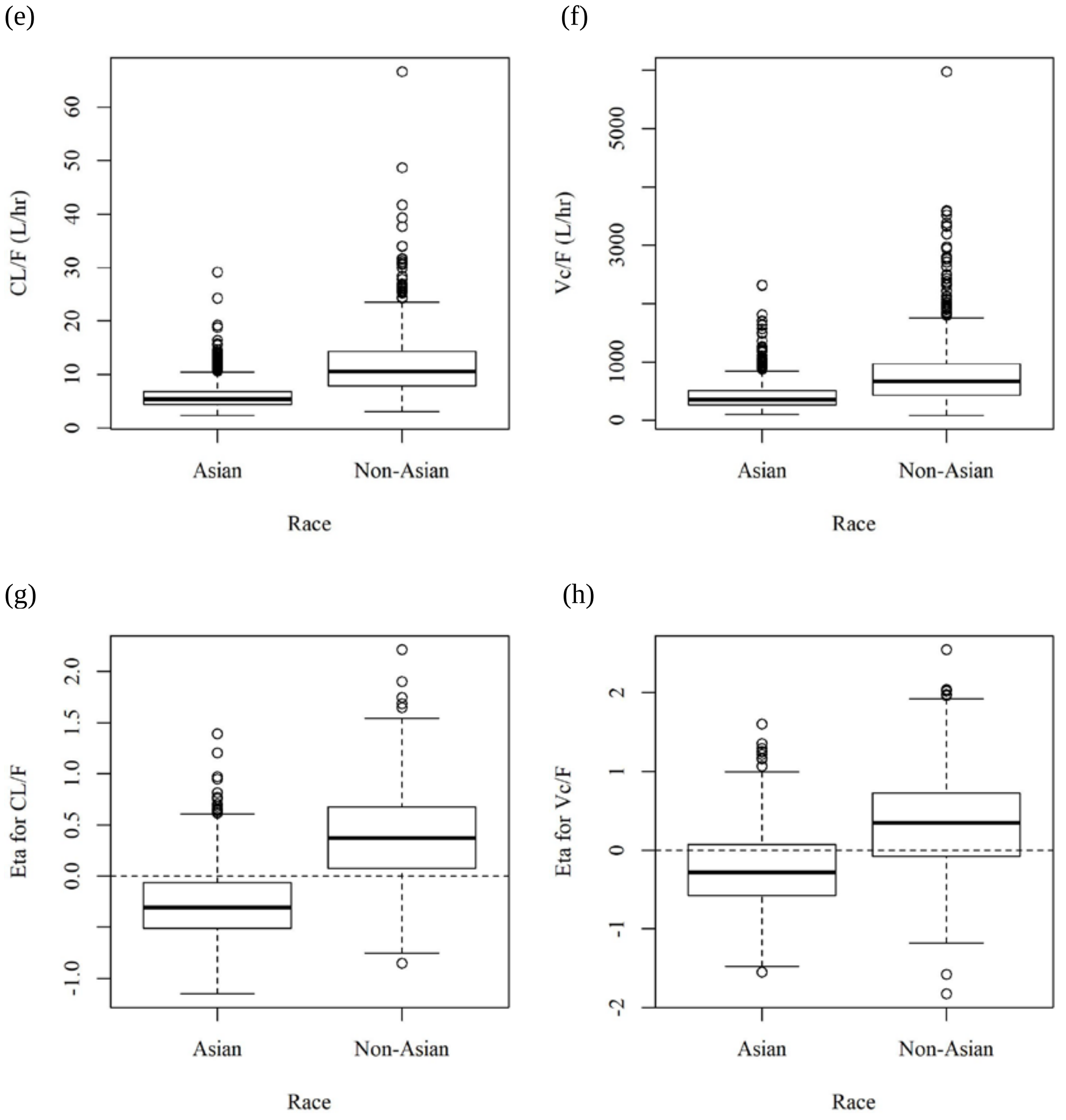
(e)
CL/F (L/hr)
0
10
20
30
40
50
60
Asian
Non-Asian
Race
(f)
Vc/F (L/hr)
0
1000
3000
5000
Asian
Non-Asian
Race
(g)
Eta for CL/F
-1.0
0.0
0.5
1.0
1.5
2.0
Asian
Non-Asian
Race
(h)
Eta for Vc/F
-2
-1
0
1
2
Asian
Non-Asian
Race

Figure S4 Goodness-of-fit plots for the final model. Plots of (a) PRED versus DV, (b) log(PRED) versus log(DV), (c) IPRED versus DV, (d) log(IPRED) versus log(DV), (e) IPRED versus absolute IWRES, (f) PRED versus CWRESI, and (g) time versus CWRESI. Red dashed line represents a locally-weighted scatterplot smoothing line.

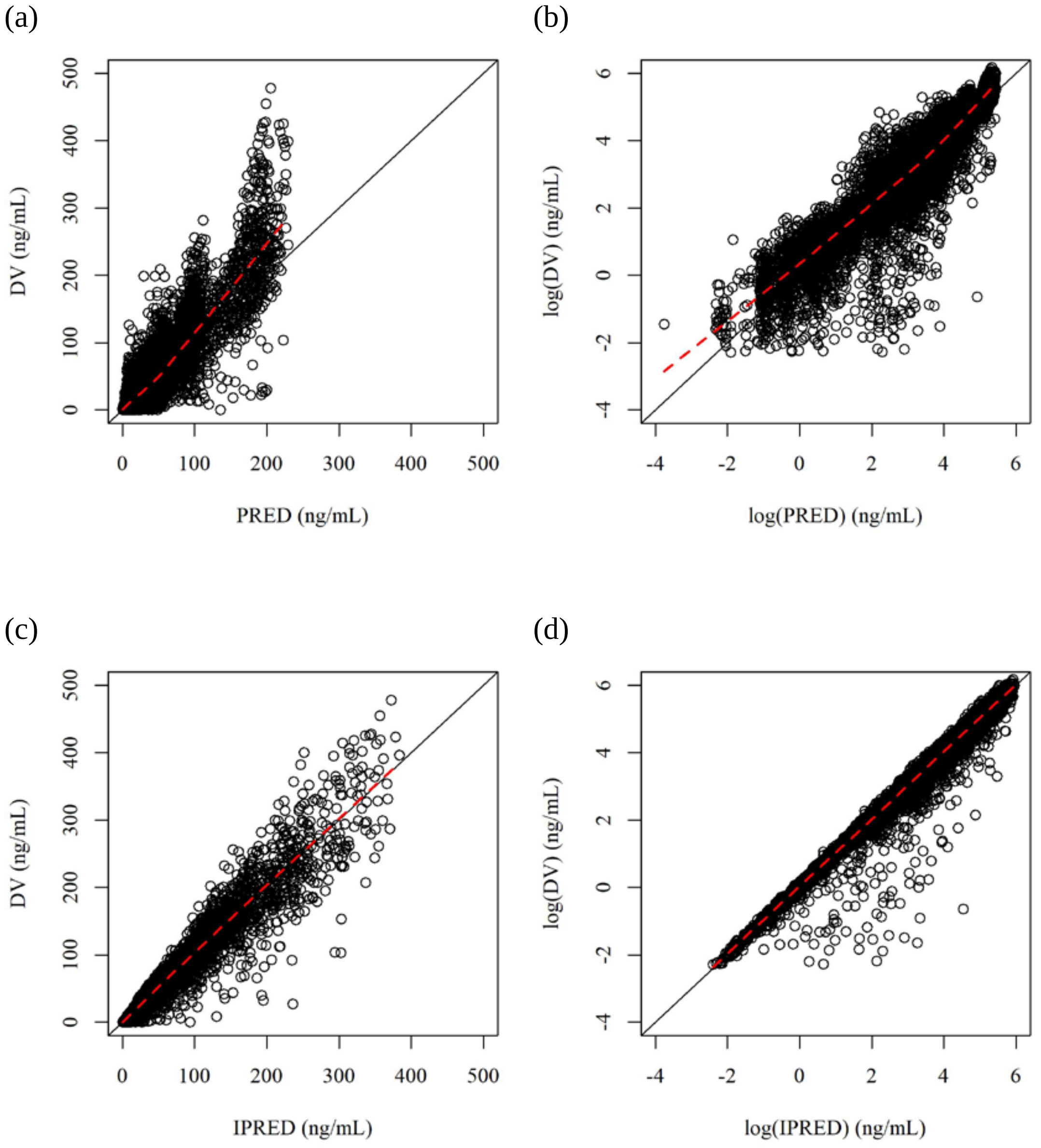

(e)

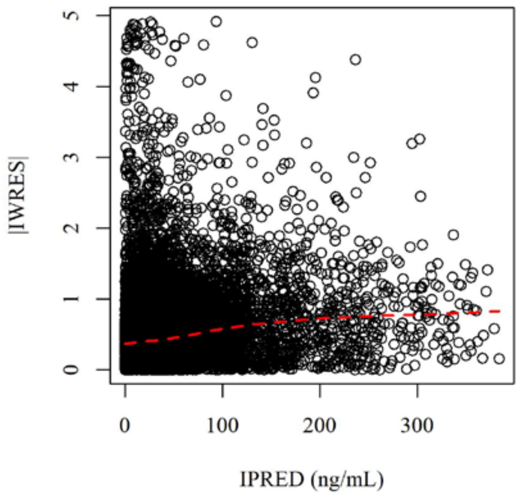
|IWRES|
0
1
2
3
4
5
0
100
200
300
IPRED (ng/mL)

(f)

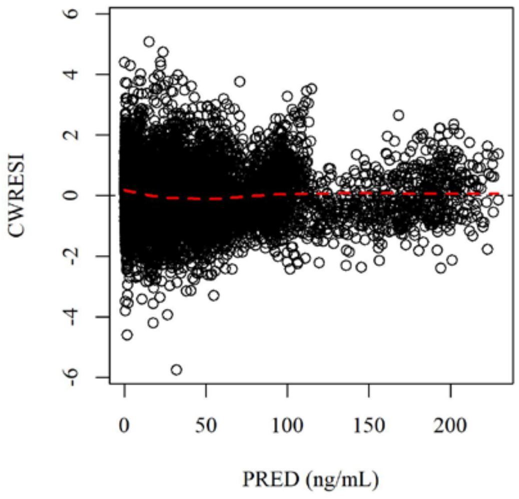
CWRESI
-6
-4
-2
0
2
4
6
0
50
100
150
200
PRED (ng/mL)

(g)

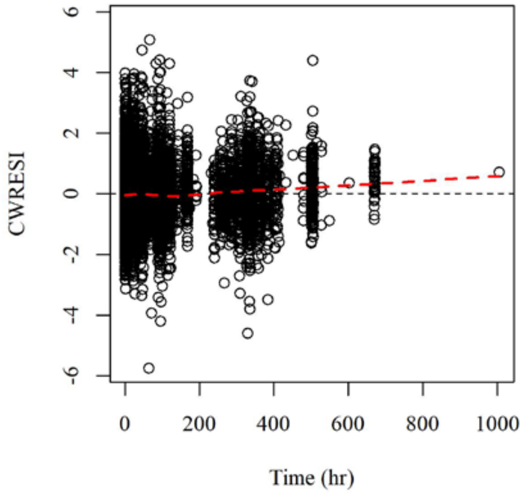
CWRESI
-6
-4
-2
0
2
4
6
0
200
400
600
800
1000
Time (hr)

Figure S5 Prediction-corrected visual predictive check. Observed plasma concentrations were compared with the observed median (solid line), 2.5th/97.5th percentiles (dotted lines), and their 95% prediction intervals (grey areas) simulated based on the final model. The plots are the observed plasma concentrations versus time after dose for overall population with observed concentrations in (a) linear and (b) semi-logarithmic axis, overall population without observed concentrations in (c) linear and (d) semi-logarithmic axis, Phase 2/3 Asian patients with observed concentrations in (e) linear and (f) semi-logarithmic axis, and for Phase 2/3 non-Asian patients with observed concentrations in (g) linear and (h) semi-logarithmic axis.

(a)

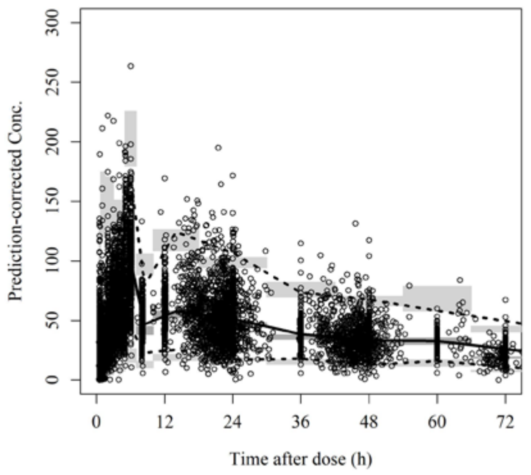

(b)

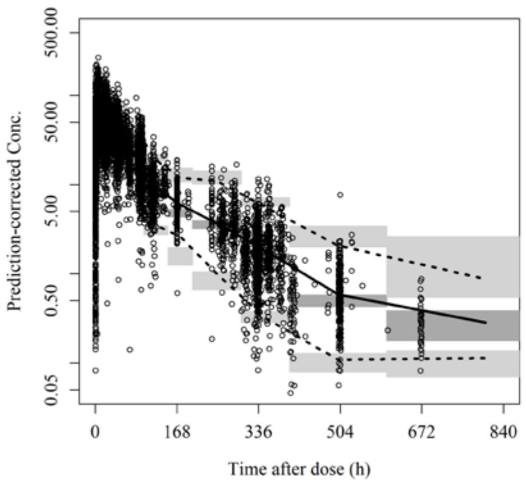

(c)

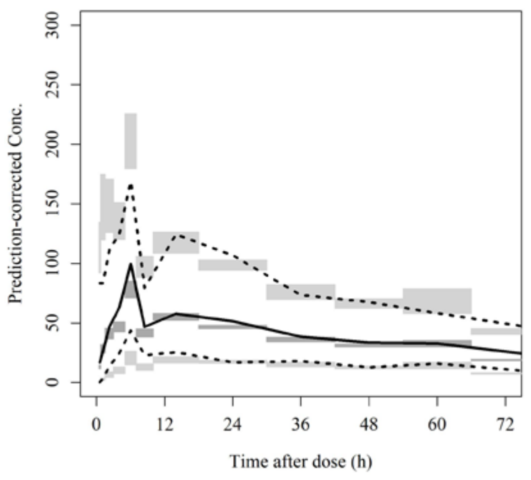

(d)

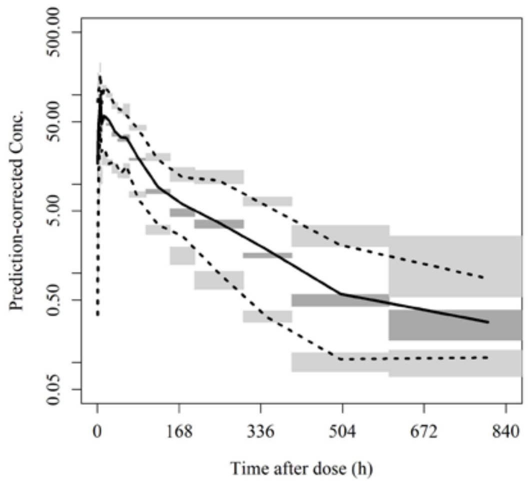

(e)

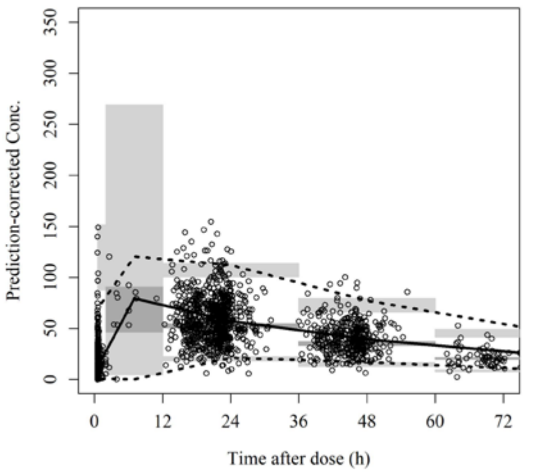
Prediction-corrected Conc.
Time after dose (h)

(f)

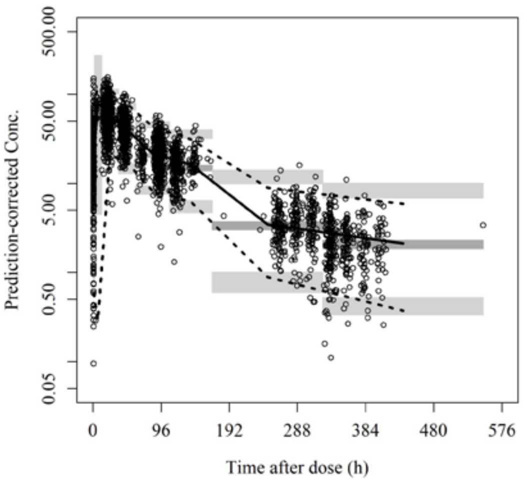
Prediction-corrected Conc.
Time after dose (h)

(g)

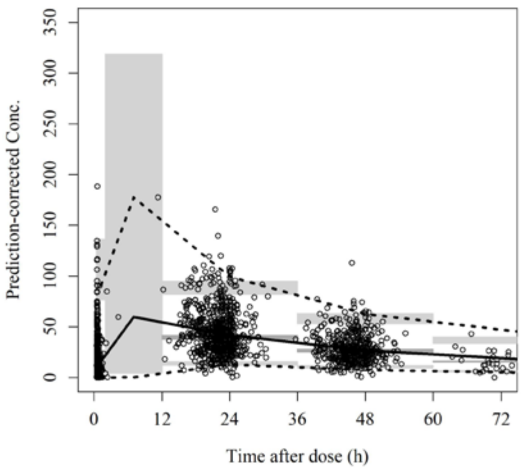
Prediction-corrected Conc.
Time after dose (h)

(h)

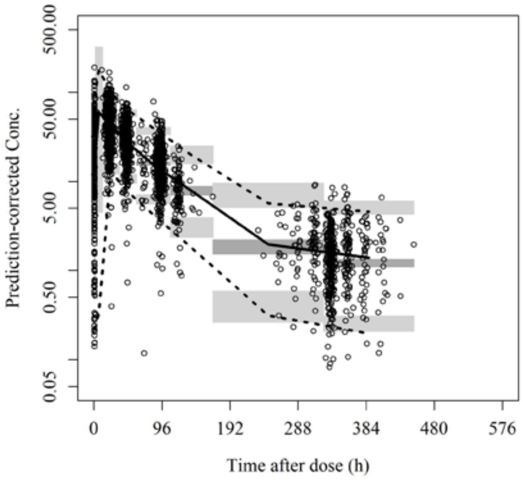
Prediction-corrected Conc.
Time after dose (h)

Figure S6 Boxplots of η for CL/F by each risk factor for the final model: (a) asthma or chronic lung disease, (b) endocrine disorders, (c) neurological and neurodevelopmental disorders, (e) heart disease, (f) at least 65 years of age, (g) metabolic disorders, and (h) morbid obesity [BMI ≥ 40]

(a)

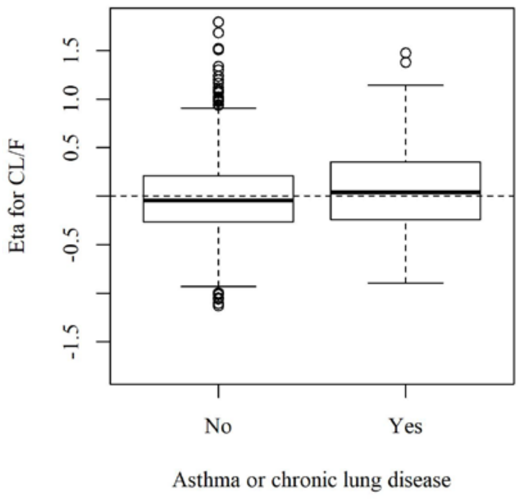

(b)

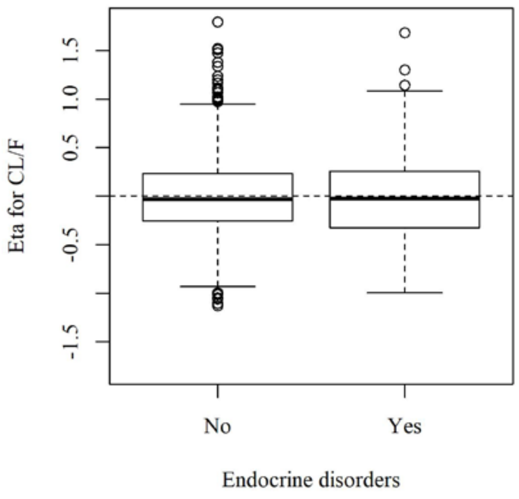

(c)

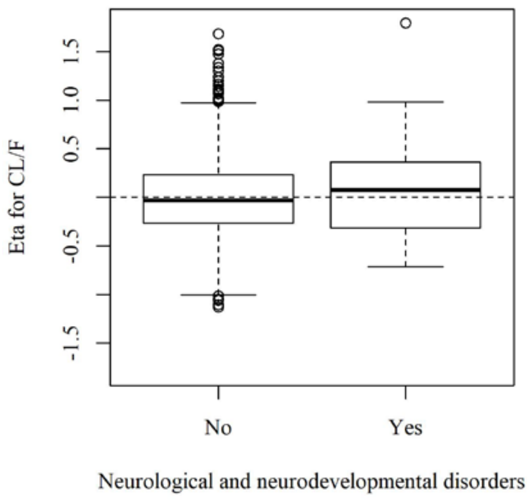

(d)

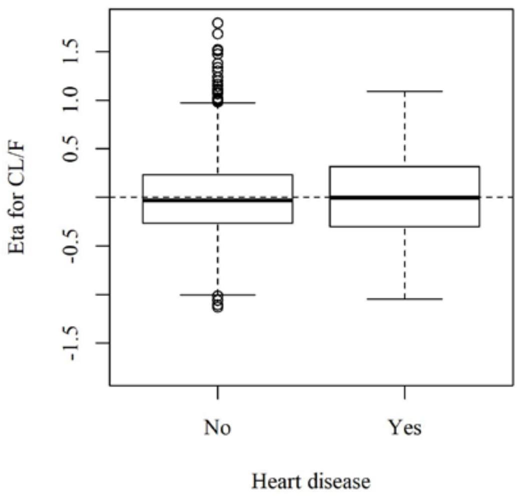

(e)

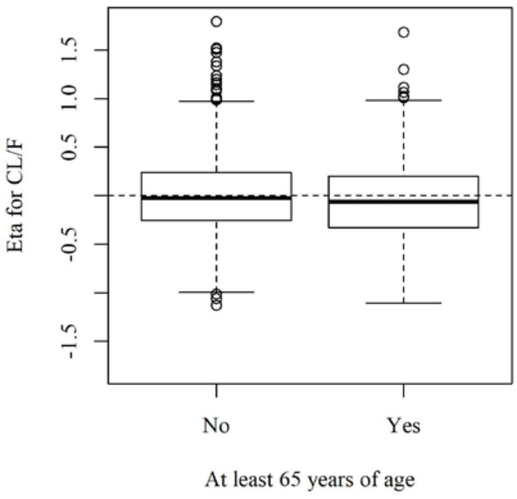
Eta for CL/F
1.5
1.0
0.5
-0.5
-1.5
No
Yes
At least 65 years of age

(f)

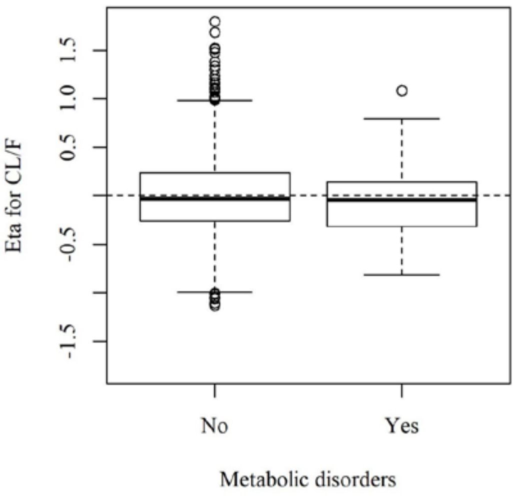
Eta for CL/F
1.5
1.0
0.5
-0.5
-1.5
No
Yes
Metabolic disorders

(g)

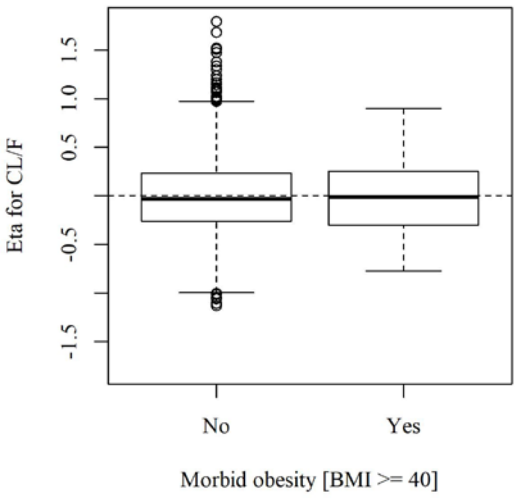
Eta for CL/F
1.5
1.0
0.5
-0.5
-1.5
No
Yes
Morbid obesity [BMI >= 40]

Figure S7 Exposure-response relationships of time to improvement of influenza symptoms versus C_{max} for (a) virus type A and (b) virus type B, AUC for (c) virus type A and (d) virus type B, and C_{24} for (e) virus type A and (f) virus type B. Red dashed line represents a locally-weighted scatterplot smoothing line.

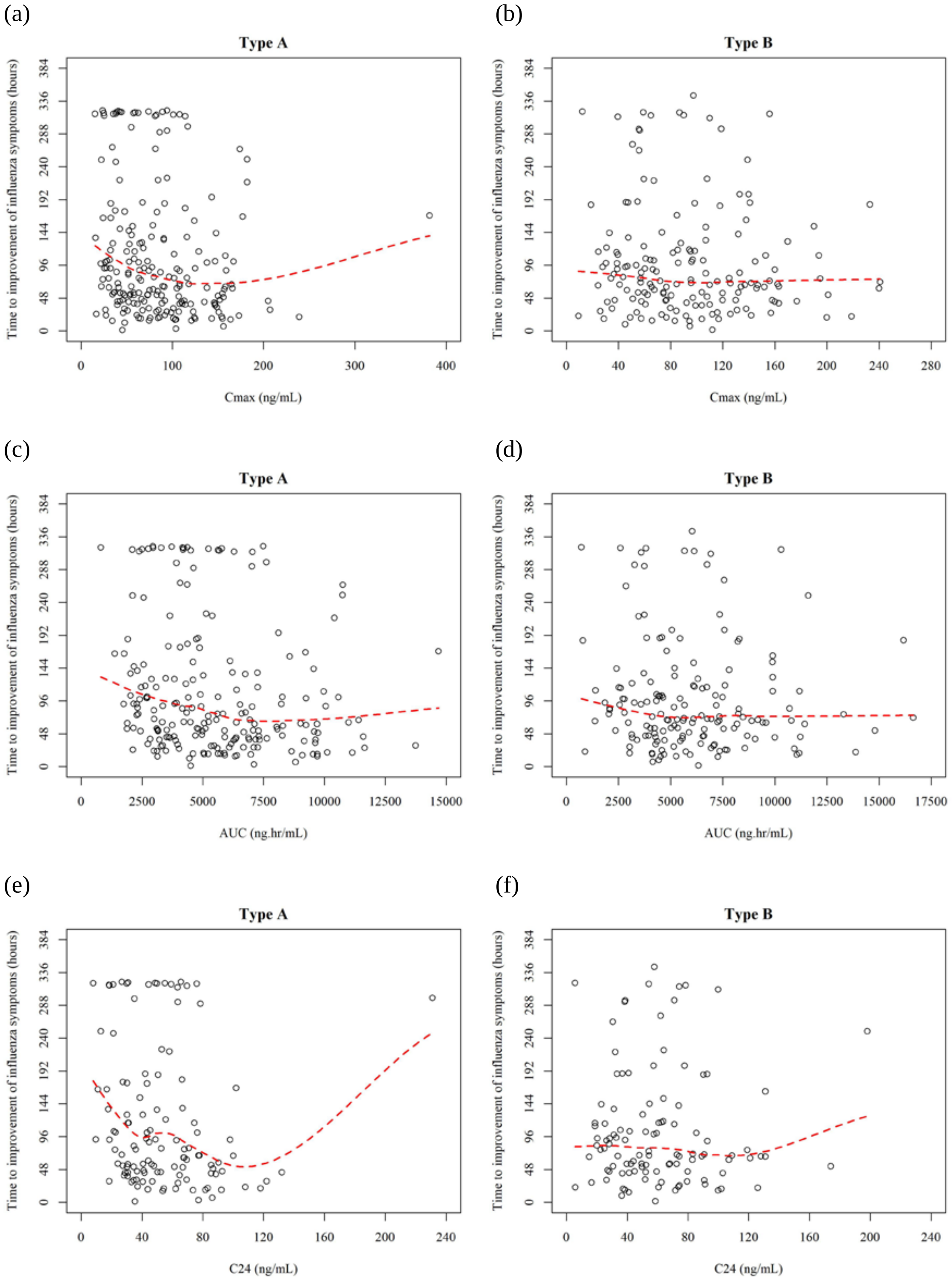

Figure S8 Exposure-response relationships of change from baseline in virus titer on day 2 versus C_{max} for (a) virus type A and (b) virus type B, AUC for (c) virus type A and (d) virus type B, and C_{24} for (e) virus type A and (f) virus type B. Red dashed line represents a locally-weighted scatterplot smoothing line.

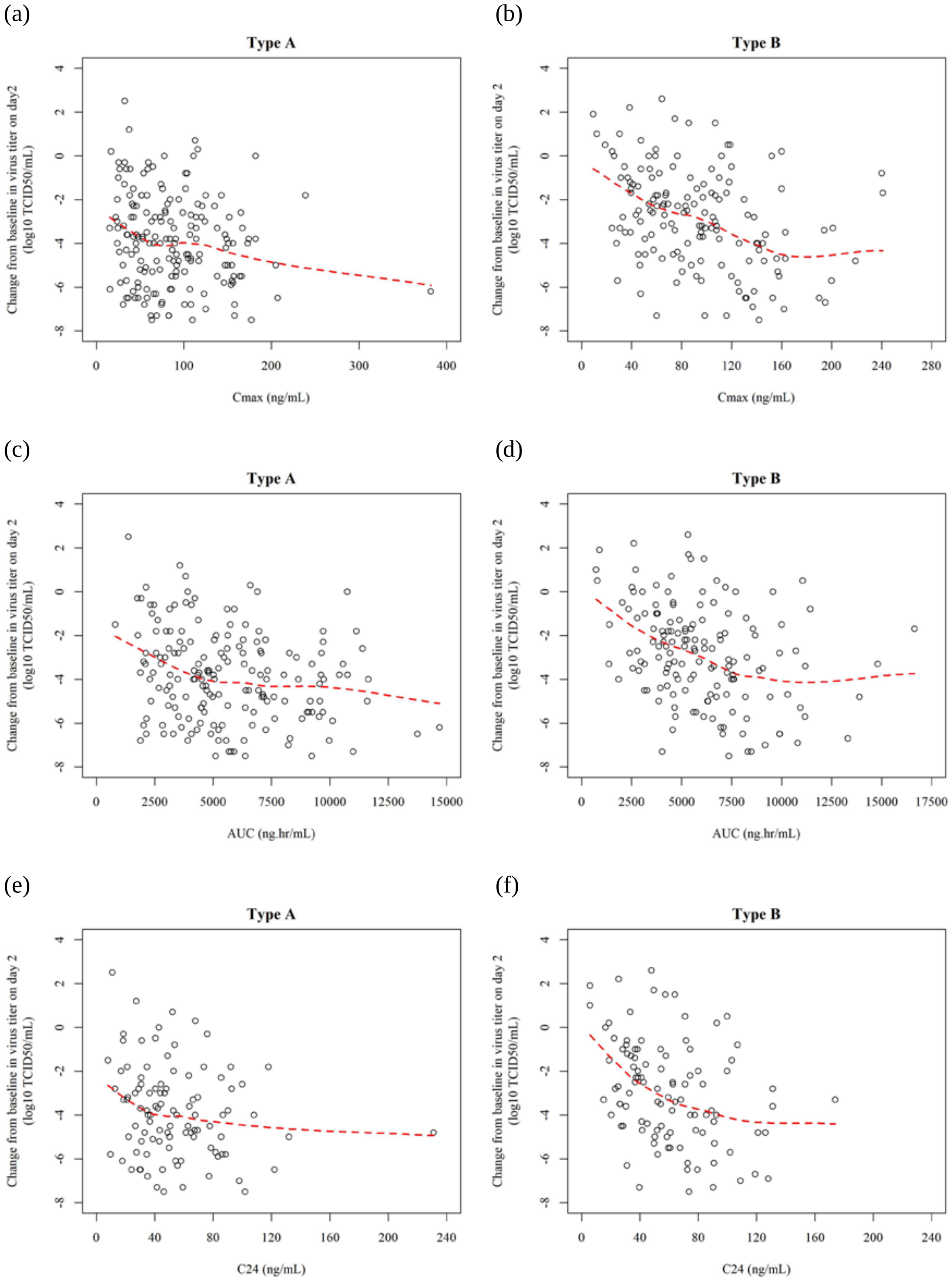

Figure S9 Relationships of time to improvement of influenza symptoms by virus type with C_{max} for (a) virus type A and (b) virus type B, AUC for (c) virus type A and (d) virus type B, and C_{24} for (e) virus type A and (f) virus type B. Thick center line represents median, top and bottom of the box represent the 1st and 3rd quartiles, and whiskers represent the 10th/90th percentile. The dashed line represents median value for placebo treatment. The number in the brackets represents subject number in each category.

(a)

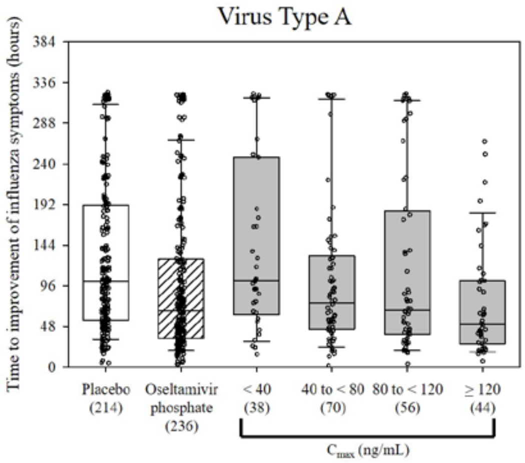

(b)

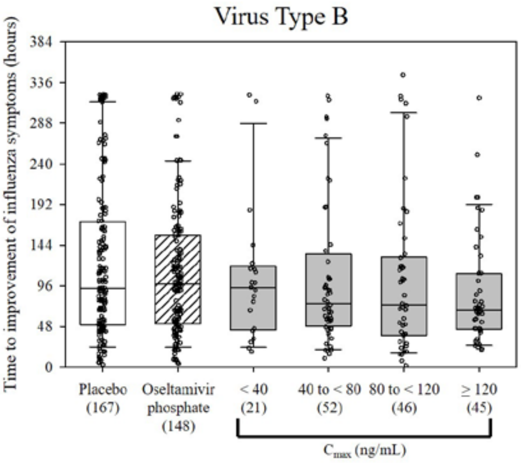

(c)

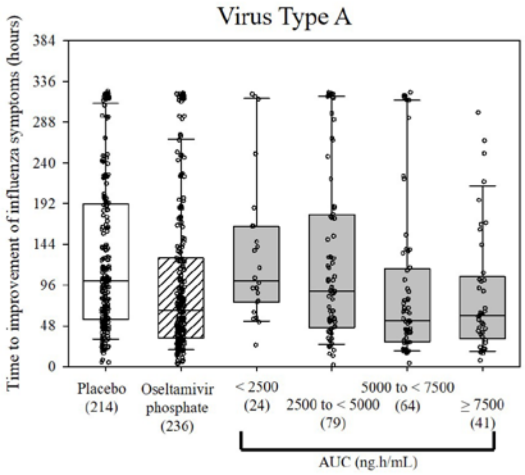

(d)

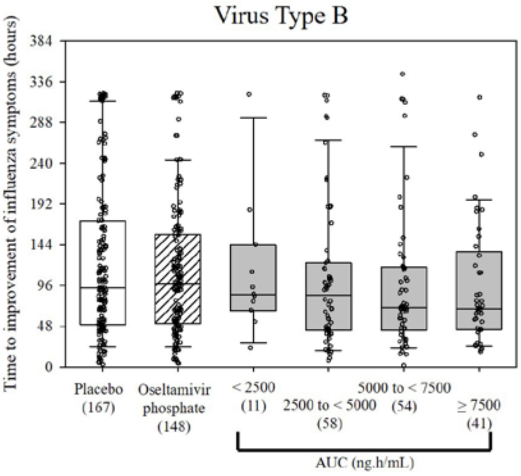

(e)

(f)

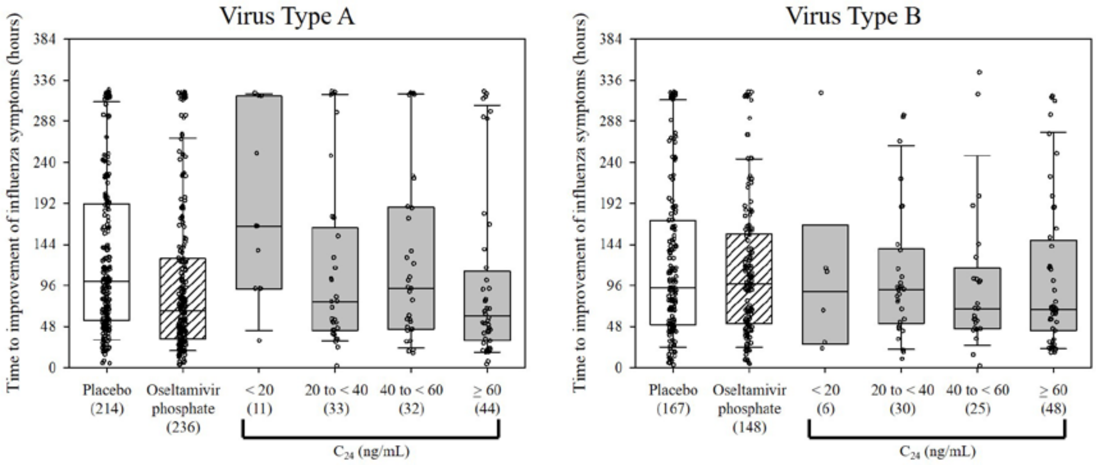

Virus Type A
Time to improvement of influenza symptoms (hours)
0
48
96
144
192
240
288
336
384
Placebo (214)
Oseltamivir phosphate (236)
< 20 (11)
20 to < 40 (33)
40 to < 60 (32)
≥ 60 (44)
C24 (ng/mL)
Virus Type B
Time to improvement of influenza symptoms (hours)
Placebo (167)
Oseltamivir phosphate (148)
< 20 (6)
20 to < 40 (30)
40 to < 60 (25)
≥ 60 (48)
C24 (ng/mL)

Figure S10 Relationships of change from baseline in virus titer on day 2 by virus type with C_{max} for (a) virus type A and (b) virus type B, and AUC for (c) virus type A (d) virus type B. Thick center line represents median, top and bottom of the box represent the 1st and 3rd quartiles, and whiskers represent the 10th/90th percentile. The dashed line represents median value for placebo treatment. The number in the brackets represents subject number in each category.

(a)

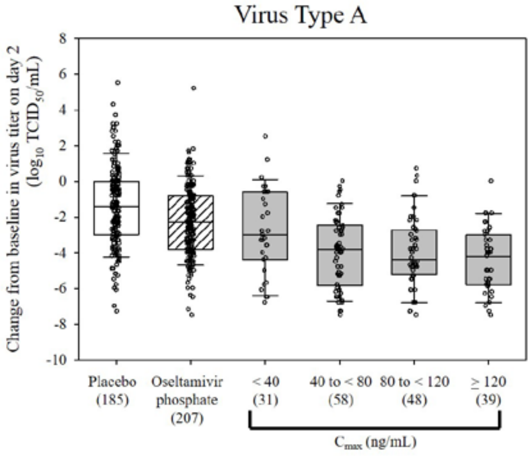

(b)

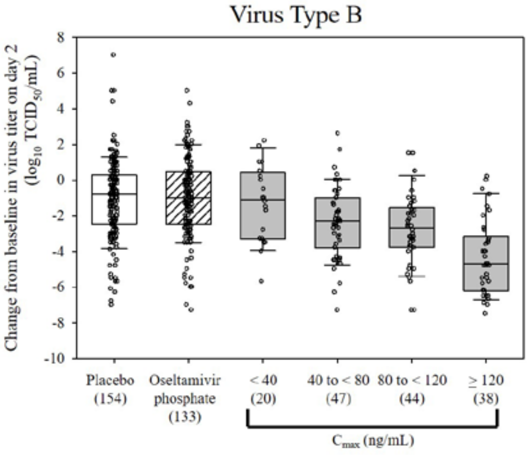

(c)

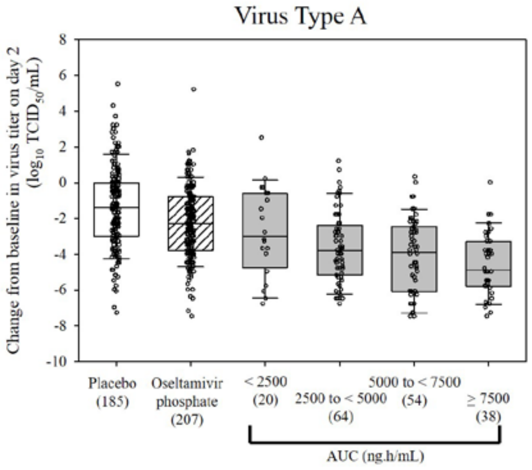

(d)

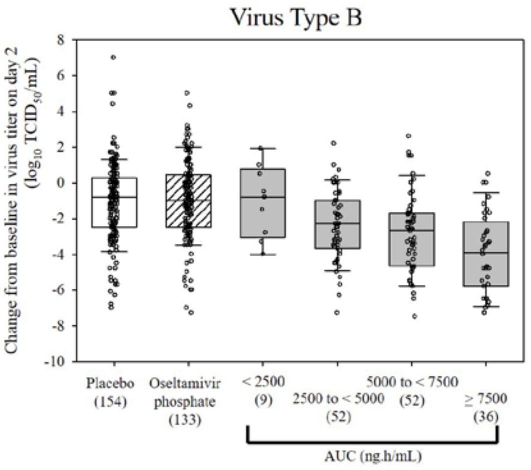

Supplement: Supplemental file 1 [file AAC.00119-20-s0001.pdf]
